# Inversed Ratio of CD39/CD73 Expression on γδ T Cells in HIV Versus Healthy Controls Correlates With Immune Activation and Disease Progression

**DOI:** 10.3389/fimmu.2022.867167

**Published:** 2022-04-22

**Authors:** Katharina Kolbe, Melanie Wittner, Philip Hartjen, Anja-Dorothee Hüfner, Olaf Degen, Christin Ackermann, Leon Cords, Hans-Jürgen Stellbrink, Friedrich Haag, Julian Schulze zur Wiesch

**Affiliations:** ^1^ First Department of Medicine, Section Infectious Diseases, University Medical Center Hamburg-Eppendorf, Hamburg, Germany; ^2^ German Center for Infection Research (DZIF), Partner Site Hamburg Lübeck Borstel Riems, Hamburg, Germany; ^3^ Department of Oral and Maxillofacial Surgery, University Medical Center Hamburg-Eppendorf, Hamburg, Germany; ^4^ Infectious Diseases Clinic, University Medical Center Hamburg-Eppendorf, Hamburg, Germany; ^5^ ICH Study Center, Hamburg, Germany; ^6^ Institute of Immunology, University Medical Center Hamburg-Eppendorf, Hamburg, Germany

**Keywords:** γδ T cells, HIV-1, T cell, CD39, CD73, Vδ2, IL-10, elite controllers

## Abstract

**Background:**

γδ T cells are unconventional T cells that have been demonstrated to be crucial for the pathogenesis and potentially for the cure of HIV-1 infection. The ectonucleotidase CD39 is part of the purinergic pathway that regulates immune responses by degradation of pro-inflammatory ATP in concert with CD73. Few studies on the expression of the ectoenzymes CD73 and CD39 on human γδ T cells in HIV have been performed to date.

**Methods:**

PBMC of n=86 HIV-1-infected patients were compared to PBMC of n=26 healthy individuals using 16-color flow cytometry determining the surface expression of CD39 and CD73 on Vδ1 and Vδ2 T cells in association with differentiation (CD45RA, CD28, CD27), activation and exhaustion (TIGIT, PD-1, CD38, and HLA-DR), and assessing the intracellular production of pro- and anti-inflammatory cytokines (IL-2, TGF-ß, TNF-α, Granzyme B, IL-10, IFN-γ) after *in vitro* stimulation with PMA/ionomycin.

**Results:**

CD39 and CD73 expression on γδ T cells were inversed in HIV infection which correlated with HIV disease progression and immune activation. CD39, but not CD73 expression on γδ T cells of ART-treated patients returned to levels comparable with those of healthy individuals. Only a small subset (<1%) of γδ T cells co-expressed CD39 and CD73 in healthy or HIV-infected individuals. There were significantly more exhausted and terminally differentiated CD39+ Vδ1 T cells regardless of the disease status. Functionally, IL-10 was only detectable in CD39+ γδ T cells after *in vitro* stimulation in all groups studied. Viremic HIV-infected patients showed the highest levels of IL-10 production. The highest percentage of IL-10+ cells was found in the small CD39/CD73 co-expressing γδ T-cell population, both in healthy and HIV-infected individuals. Also, CD39+ Vδ2 T cells produced IL-10 more frequently than their CD39+ Vδ1 counterparts in all individuals regardless of the HIV status.

**Conclusions:**

Our results point towards a potential immunomodulatory role of CD39+ and CD73+ γδ T cells in the pathogenesis of chronic HIV infection that needs further investigation.

## Introduction

The human immunodeficiency virus-1 (HIV) is a lymphotropic virus that mainly infects and depletes CD4+ T cells, leading to chronic immune activation, immune dysfunction, and, ultimately, immunodeficiency (1–4). Although highly active antiretroviral therapy (HAART) potently suppresses viral replication, no cure is available to date (5, 6).

Lately, the role of unconventional T cells for HIV pathogenesis and HIV cure approaches has come more into focus (7–11). Among these T-cell populations, γδ T cells seem to have important immunomodulatory properties relevant for the disease (4). γδ T cells express a T-cell receptor with gamma and a delta chain (12–17). They are “innate-like” T cells that make up 1-15% of circulating leukocytes and exert a direct cytotoxic activity independently of MHC presentation (12–22). About 30% of γδ T-cells express a CD8+ T cell receptor, less than 1% a CD4+ T-cell receptor and 70% none of the conventional T-cell receptors (23).

γδ T cells recognize stress-induced molecules, non-peptide, and phosphoantigens, self- or MHC-related molecules, and lipids associated with different kinds of pathogens (20, 21, 24–29). γδ T cells are generally seen to be pro-inflammatory and involved in the initiation and propagation of immune responses, but lately, it has been shown that they also act as immunomodulators and can inhibit T- and B-cell responses (20, 21, 30–32). They produce the pro-inflammatory cytokines TNF-α, IL-17, and IFN-γ as well as the anti-inflammatory cytokines IL-10 and TGF-β and IL-2, IL-22, IL-21, IL-4, IL-5, IL-13 (31, 33–38). γδ T cells can alleviate or maximize inflammation in the blood and different tissues and might be used to directly target HIV-infected cells but have also been described as potential targets of HIV (18, 22, 39, 40).

The two main subsets of γδ T cells, Vδ1 and Vδ2 are present in different anatomic compartments (23, 41–44). While Vδ1 cells can be found in the intraepithelial layer of mucosal surfaces, the Vδ2 population is mostly present in the blood and secondary lymphoid tissues of healthy adults (ratio in the peripheral blood Vδ1:Vδ2 3:10) (23, 41–49).

Early during primary HIV infection, an inversion of the Vδ1:Vδ2 ratio can be observed in the blood whereas the frequency of total γδ T cells remains relatively stable (18, 22). Vδ2 cells are depleted while Vδ1 cells expand (4, 18, 50–52). It has been shown that Vδ2 cells express high levels of the HIV co-receptors CCR5 and α4β7, which possibly contributes to their preferential depletion in HIV infection (53–56). Their number, but also functionality remains below that of healthy controls even after successful implementation of ART and restoration of the CD4+ T-cell compartment (51, 52). It has been implied that Vδ1 cells are involved in antiviral immunity and their expansion may be an indirect consequence of viral infection and reflects an increased translocation of stimulatory bacterial products across the gut epithelium in non-human primate studies (57–59). In contrast, direct cytotoxicity towards HIV-infected cells has been demonstrated to be largely restricted to Vδ2 cell clones, and the frequency of cervical Vδ2 cells correlates with SIV viral load (60–62). Interestingly, elite controllers exhibit higher frequencies of Vδ2 cells than untreated or antiretroviral treated HIV progressors (63, 64).

The ectonucleotidases CD39 and CD73, members of the adenosine pathway that are expressed on several lymphocyte populations, convert extracellular pro-inflammatory ATP and ADP to anti-inflammatory Adenosine (ADO) (65–67). In healthy individuals, the level of extracellular ADO is low but can increase 100-1000-fold in situations of strong inflammation and tissue injury (56). ADO strengthens epithelial barrier functions and inhibits leukocyte extravasation by binding to four different receptors: A1R, A2AR, A2BR, and A3R (68, 69). ADO binding to A2AR increases intracellular cAMP and inhibits the production of cytokines and T-cell proliferation (70–74). Importantly, both CD39 and CD73 can work in cis (interaction on the same cell), trans (interaction with enzymes expressed on different cells), and as soluble forms, and detailed knowledge about the respective microenvironment is essential (67, 75).

Over the last decade, multiple roles of CD39 and CD73 in the regulation of inflammation and immune responses have been revealed (75–82). It has also been demonstrated that mutations in the purine system can cause severe primary immunodeficiency diseases (78, 83–85). In HIV-infected untreated individuals, an over-expression of CD39 on and an increased hydrolysis of ATP by lymphocytes has been observed. Also, a variant of the CD39 gene associated with low CD39 expression and a slower progression to AIDS has been described in lymphocytes (86–88). Furthermore, the CD39/CD73/adenosine axis has been linked to inhibition of HIV-1 replication as well as immune suppression by CD39+ regulatory T cells (Tregs) (66, 88, 89).

In viremic HIV patients, the frequency of CD73+ cells in different T-cell subsets, especially Tregs and CD8+ T cells, is markedly reduced, and the function of CD73+ CD8+ T cells is impaired (82, 90). On B cells, low CD73 and CD39 expression are associated with low CD4+ T-cell counts (77). While both CD39 and CD73 are co-expressed on murine Tregs, only a small fraction of human peripheral Tregs expresses CD73 (65, 66, 82, 91).

Higher CD73 levels have been associated with immunosuppression and poor prognosis in e.g. breast or ovarian cancer (76, 92–95). In mice, suppressive activities of CD73+ γδ T cells *via* adenosine were shown (96).

Only recently it has been shown that γδ T cells can also act in an immunosuppressive manner and that they can infiltrate tumors and suppress dendritic cells and T cells (30, 97, 98). Liang et al. demonstrated that the regulation of γδ T cells in autoimmunity is associated with ADO (96). Hu et al. described CD39^+^ γδ T cells as capable of suppressing T cells *via* the adenosine-mediated pathway but independent of IL-10 and TGF-beta expression (98). In contrast, Otsuka et al. have reported a potential role of CD39+ γδ T cells with a regulatory phenotype mediated by IL-10 secretion in mice (98, 99).

In HIV infection, the plasma concentration of IL-10 increases over time and limits specific T-cell responses (100). CD39+ NK cells secreting IL-10 also contribute to this increase: Dierks et al. demonstrated that elevated levels of CD39+ NK cells in viremic patients correlated directly with viral load and activation, and negatively correlated with CD4+ T-cell count (101). IL-10 secretion was associated with the expression of CD39 (99, 101–103).

We and others have previously shown that CD39 expression of Tregs correlates with the progression of HIV infection and that Tregs of HIV elite controllers show the lowest levels of CD39 (79, 104). We have also recently identified CD39+ γδ T cells with an immunosuppressive phenotype in the gut (81). Bhatnagar et al. suggested a suppressive activity especially of Vδ2 *via* TGF-β, which is dysregulated in progressed HIV infection (35).

In HIV infection, a comprehensive assessment of the expression of CD39 and CD73 on different γδ subsets including Vδ1 and Vδ2 γδ T cells has never been performed. Therefore, we sought to characterize CD39+ and CD73+ expression on γδ T cells in relation to phenotype and function in a large cohort of healthy individuals and people living with HIV with different disease statuses including HIV elite controllers and long-term non-progressors.

## Material And Methods

### Study Subjects and Samples

Peripheral blood mononuclear cell (PBMC) samples of chronic, treatment-naïve HIV patients (viremic, n=36), HIV antiretroviral therapy (ART)-treated patients (ART, n=32), HIV elite controllers (EC, n=8), HIV long-term non-progressors (LTNP, n=10) and HIV negative healthy controls (n=26) were collected at the University Medical Center Hamburg-Eppendorf. HIV elite controllers were defined as HIV-infected individuals capable of spontaneously controlling HIV infection (maintaining stable CD4+T-cell counts and viral loads below the level of detection) without the need for antiretroviral medication (105–107). Written informed consent was obtained from all patients who were recruited for this study, which was approved by the local Institutional Review Board of the Ärztekammer Hamburg, Germany (MC-316/14, PV4780, PV5798, PV4081, WF14-09). Active Hepatitis C virus and Hepatitis B virus co-infections were ruled out serologically in the HIV-infected patients studied. CD4^+^ T-cell counts and plasma viral loads were extracted from the clinical database (**Table 1**). Clinical and virologic data of the HBV and HCV patients can be found in **Supplementary Table 1**.

**Table 1:** Demographic, virologic, and immunological basic data of the cohort [**average** (± SD) min – max].

	*Healthy*	*ART*	*Viremic*	*EC*	*LTNP*
***n***	**26**	**32**	**36**	**8**	**10**
***Sex in % (f/m/d)***	**63/37/0**	**23/77/0**	**22/78/0**	**67/33/0**	**38/62/0**
***Age (years)***	**29,1** ( ± 10,8)20- 61	**44,5** ( ± 14,3) 25 – 75	**39,6** ( ± 12,5) 22 - 72	**43,5** ( ± 14,9) 21 - 56	**47** ( ± 11,9) 32 - 73
***CD4+ T-cell count (cells/mL)***	**>500**	**531,9** (± 256,1) 125 – 1190	**276,5** ( ± 300,8) 6 - 1731	**866,3** (± 301,4) 458 - 1219	**596,4** ( ± 408) 175 - 1485
***Viral load (copies/mL)***	**n.a.**	**<10**	**312256** ( ± 566792) 6300 - 3300000	**<10**	**1323,75** ( ± 973,7) 40 - 2600

For the study, 26 healthy volunteers, 36 virally suppressed HIV patients on ART, 32 HIV-infected viremic individuals, 8 aviremic elite controllers, and 10 long-term non-progressors were included. Mean values in bold type. N.a. not applicable.

### Immune Phenotypic Analysis for Surface and Intracellular Markers

Cryopreserved PBMC were isolated and used for immunophenotypic staining as previously described (108). Cells were stained with Zombie NIR fixable viability stain (BioLegend, San Diego, USA) and the following anti-human monoclonal fluorochrome-conjugated antibodies: anti-CD45RA, anti-CD4, anti-TCR-γ/δ (BD Biosciences, Heidelberg, Germany), anti-TCR-Vδ2 (Beckman Coulter Life Sciences, Indianapolis, USA), anti–HLA-DR, anti-CD27, anti-CD279 (PD-1), anti-TIGIT, anti-CD8, anti-CD28, anti-CD39, anti-CD38, anti-CD19, anti-CD3, anti-CD73 and anti-CD14 (all BioLegend) (**Supplementary Table 2**). Cells were incubated for 30 minutes at room temperature with the respective antibodies. After washing, cells were fixated with 4% paraformaldehyde. All samples were run on a Becton Dickinson LSR Fortessa flow cytometer with FACS Diva version 8 (BD Biosciences).

### Intracellular Cytokine Staining and Kinetic of CD39 Expression After *In Vitro* Stimulation of PBMC

For intracellular staining, cells were stimulated with phorbol 12-myristate 13-acetate (PMA; final concentration 25 ng/mL; Merck, Darmstadt, Germany) and ionomycin (final concentration 1 µg/mL; Merck) for 18 hours. After 2 hours, Brefeldin A (5µg/mL; Merck) and Monensin (1µg/mL; Merck) were added. First, surface antigens were stained as described above. Cells were then permeabilized with fixation/permeabilization solution (Cytofix/Cytoperm; BD Biosciences) and stained with fluorochrome-conjugated antibodies for 30 minutes at 4°C. The following anti-human monoclonal antibodies were used: anti-IL-2, anti-CD4, anti-IL-10, anti-TCR-γ/δ (BD Biosciences), anti-TCR-Vδ2 (Beckman Coulter Life Sciences), anti–IFN-γ, anti-TNF-α, anti-CD8, anti-TGF-β, anti-CD39, anti-Granzyme-B, anti-CD19, anti-CD3, anti-CD73, and anti-CD14 (all BioLegend), see also **Supplementary Table 3**.

For kinetic studies of CD39 surface expression, cells were stimulated as previously described with small adaptations (109). Briefly, cryopreserved PBMC were plated into 48-well plates and stimulated with rhIL-2 (20 U/mL; Miltenyi Biotec, Bergisch Gladbach, Germany), PMA (5 ng/mL), ionomycin (0,5 µg/mL), anti-CD3/CD28-Dynabeads (ratio 1:1; ThermoFisher Scientific, Waltham, USA) or combinations thereof. Cells were cultured for up to 6 days before FACS analysis.

### Data Analysis and Statistics

Cytometric data were analyzed using FlowJo version 10.7.1 (BD Biosciences). The applied gating strategy and exemplary dot plots are depicted in **Supplementary Figures 2**, **3** and **8**. Statistical analysis was performed using GraphPad Prism version 7.04 (GraphPad Software, Inc., La Jolla, CA). For multiple comparisons, Kruskal–Wallis and Dunn’s post-test with an alpha value of 0.05 were performed. All reported *P* values were multiplicity adjusted according to Dunn. To compare ranks, 2-tailed Mann–Whitney and Wilcoxon tests were performed. Pearson correlation and Spearman rank correlation coefficient were applied for bivariate correlation analysis. Multidimensional cytokine analysis was carried out using SPICE 6 (110). Data are expressed as mean with SD. P-values of less than 0,05 were considered significant. Levels of significance correspond to asterisks as follows: ns p≥0,05; * p<0,05; ** p≤0,01; *** p≤0,001; **** p≤0,0001.

## Results

The ectonucleotidases CD39 and CD73 have been described as important immunoregulatory molecules on Tregs and T effector cells (67, 75–79, 81, 82, 92, 93, 98, 99, 101, 104, 111). In mice, CD39+ γδ T cells with a regulatory phenotype have already been described (99, 112). In humans, CD39+ immunosuppressive γδ T cells have been described in the context of colon cancer (98). Little is known about the expression of these two molecules on γδ T cells in healthy humans and the context of viral infections. In this study, we aimed at the detailed assessment of the CD39 and CD73 expression pattern on peripheral γδ T cells in healthy and HIV-infected individuals with respect to their differentiation, activation, and exhaustion status and their immunomodulatory properties in terms of their cytokine profiles.

In line with previously published data, we found that the percentage of total γδ T cells was stable during HIV infection regardless of the stage of HIV infection while the ratio between the subsets Vδ1 and Vδ2 was inversed (**Supplementary Figure 1** **;** see **Supplementary Figures 2** and **3** for the gating strategy) (4, 18, 22, 40). In healthy individuals, the percentage of Vδ2 (68,5%) was significantly higher than that of Vδ1 γδ T cells (31,4%, p=0,0027), whilst it was significantly lower in viremic (Vδ1: 81,6%, Vδ2: 18,4%, p<0,0001) and patients on ART (Vδ1: 73,3%, Vδ2: 26,8%, p<0,0001). Interestingly, in long-term non-progressors, the proportions of Vδ1 (50,34%) and Vδ2 γδ T cells (49,7%) were similar. In elite controllers, we observed a difference between Vδ1 (69,7%) and Vδ2 (30,3%) γδ T-cell proportion that did not reach statistical significance, most likely due to the small patient number.

### The Frequency of CD39+ γδ T Cells Increases While the Frequency of CD73+ γδ T Cells Decreases in HIV Infection

**Figure 1:**
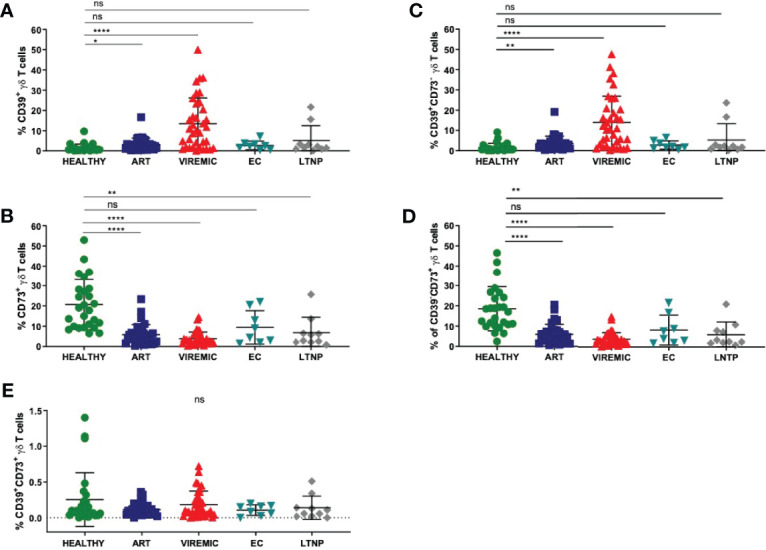
The relative frequency of CD39+ γδ T cells is increased in viremic and HIV patients on ART compared to healthy controls **(A)** while the frequency of CD73+ γδ T cells is decreased in HIV infection regardless of the disease status **(B)**. **(C)** Frequency of CD39+CD73- γδ T cells in PBMC. **(D)** Frequency of CD39-CD73+ γδ T cells in PBMC. **(E)** Frequency of double-positive CD39+CD73+ γδ T cells in PBMC. Data from 26 healthy individuals, 32 viremic, 36 HIV patients on ART, 8 EC, and 10 LTNP. ns, non-significant p≥0,05; *p<0,05; **p≤0,01; ****p≤0,0001.

To understand the biology of CD39 and CD73 on γδ T cells, we first globally assessed the expression pattern of the two ectonucleotidases on total γδ T cells in healthy individuals and HIV patients who were further sub-stratified according to their disease status (**Figures 1A, B** **)**. The highest frequency of CD39+ γδ T cells was detected in samples from viremic HIV-infected patients. In viremic and individuals on ART, the CD39+ γδ T-cell frequency was significantly increased compared to healthy individuals (viremic: 11,3% vs. 1,4%, p<0,0001; ART: 3,2% vs. 1,4%, p=0,0146). Of interest, in samples from EC, the frequency of CD39+ γδ T cells was similar compared to samples from healthy controls (2,7%), while it was slightly elevated in LTNP (5,1%) compared to healthy controls. The differences between CD39+ γδ T cells in EC/LTNP and healthy controls did not reach statistical significance.

Conversely, the frequency of CD73+ γδ T cells was markedly decreased in PBMC from HIV-infected individuals regardless of their infection status compared to healthy individuals (**Figure 1B**). The differences between CD73+ γδ T cells from healthy and HIV-infected individuals were statistically significant (healthy: 20,7% vs. ART: 5,8%, p<0,0001; viremic: 3,7%, p<0,0001; LTNP: 6,8%, p=0,0016). Of note, only the differences between CD73+ γδ T cells from healthy individuals and EC were non-significant (EC: 9,4%, p=0,0525).

Next, we analyzed the co-expression pattern of CD39 and CD73 on γδ T cells. The number of CD39+CD73- γδ T cells was similar to the number of CD39+ γδ T cells in healthy individuals and all HIV patient subgroups (**Figures 1A, C** **)**. Also, the frequency of CD73+CD39- γδ T cells (**Figure 1D**) was similar to the frequency of CD73+ γδ T cells (**Figure 1B**). In contrast to that, the frequency of double-positive CD39+CD73+ γδ T cells was considerably lower in PBMC from all study groups and did not differ significantly between healthy and HIV-infected individuals (healthy: 0,25%; ART: 0,11%; viremic: 0,15%; EC: 0,11%; LTNP: 0,14%) (**Figure 1E**).

To understand if the pattern of CD39 and CD73 expression on γδ T cells was similarly affected in other acute or chronic viral infections, PBMC from patients with acute and chronic hepatitis B (HBV) and chronic hepatitis C (HCV) were also analyzed (**Supplementary Figure 4** and **Supplementary Table 1**). In acute HBV, an increase of CD39+ CD8+ T cells (data not shown) and a decreased frequency of CD73+ γδ T cells could be measured compared to healthy controls (7,8% vs. 23,9%). By contrast, there was no increase of CD39+ γδ T cells in patients with acute HBV, chronic HBV, or chronic HCV compared to healthy individuals. The reasons for the specific expansion of CD39+ γδ T cells in HIV compared to other viral infections are unclear and must be elucidated.

### CD39 Expression on γδ T Cells From HIV-Infected Individuals Correlates With Viral Load, CD4+ T-Cell Counts, and Immune Activation

**Figure 2:**
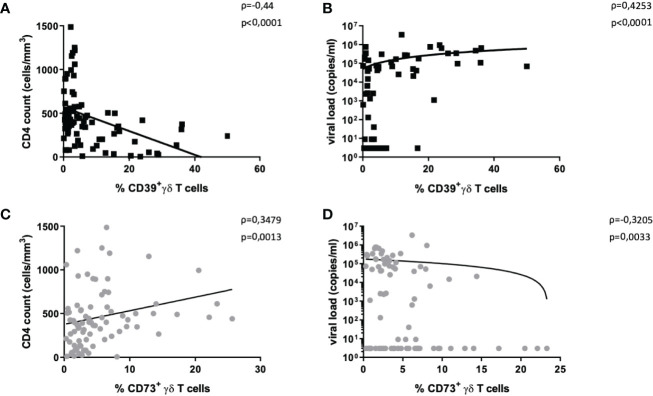
Correlation of CD39+ and CD73+ γδ T cells with disease progression markers. The frequency of CD39+ γδ T cells in HIV correlates negatively with CD4+ T-cell count **(A)** and positively with HIV viral load **(B)**. The frequency of CD73+ γδ T cells in HIV correlates positively with CD4+ T-cell count **(C)** and negatively with HIV viral load **(D)**. Graphs contain pooled data from HIV-infected patients including viremic and individuals on ART, EC, and LTNP.

Since we observed divergent expression of CD39 and CD73 on γδ T cells in PBMC from HIV-infected patients compared to healthy individuals, we next examined whether there was a correlation with standard clinical parameters defining the HIV disease course. Indeed, the frequency of CD39+ and CD73+ γδ T cells was significantly correlated with the HIV viral load and CD4+ T-cell counts (**Figure 2** **)**. Furthermore, the frequency of CD39+ γδ T cells correlated with immune activation indicated by the co-expression of HLA-DR and CD38 on CD8+ and total γδ T cells (**Supplementary Figures 5** and **6**).

There was a negative correlation between the frequency of CD39+ γδ T cells and CD4+ T-cell counts (Spearman ρ=-0,44, p<0,0001, **Figure 2A**) and a positive correlation between plasma viral load and the frequency of CD39+ γδ T cells (Spearman ρ=0,43, p<0,0001, **Figure 2B**).

The same analyses for CD73+ γδ T cells yielded the opposite results: the frequency of CD73+ γδ T cells positively correlated with CD4+ T-cell counts (Spearman ρ=0,35, p=0,0013, **Figure 2C**) and negatively with viral load (Spearman ρ=-0,32, p=0,0033, **Figure 2D**).

In samples from individuals with HIV, the frequency of CD39+ γδ T cells correlated with the proportion of activated CD8+ T cells (Spearman ρ=0,26, p=0,0375; **Supplementary Figure 5A**) and activated γδ T cells (Spearman ρ=0,42, p=0,0004; **Supplementary Figure 6**).

Interestingly, in PBMC of healthy individuals, there were non-significant negative correlations between activated CD8+ T cells and the frequency of CD39+ γδ T cells (Spearman ρ=-0,30, p=0,2205) and between activated γδ T cells and CD39+ γδ T cells (Spearman ρ=0,26, p=0,2819; **Supplementary Figures 5B** and **6**). Regardless of the disease status, a significantly higher frequency of activated cells was measured among CD39+ compared to CD39- γδ T cells (**Supplementary Figure 5C** **).** There was no correlation between the frequency of CD73+ γδ T cells and activated γδ or activated CD8+ T cells in HIV-infected patients (data not shown).

Of note, the frequency of CD39+ γδ T cells increased steadily for 6 days after *in vitro* stimulation with CD3/CD28 or PMA/ionomycin (**Supplementary Figure 7**).

Taken together, the frequency of activated CD8+ and γδ T cells correlated with the frequency of CD39+ γδ T cells in HIV infection, and CD39+ γδ T cells expanded in response to *in vitro* stimulation.

### Vδ2 γδ T Cells Are Less Exhausted and Less Differentiated Than Their Vδ1 γδ T-Cell Counterparts But Do Not Differ in Their Activation Status

Vδ1 and Vδ2 T cells differ considerably in their phenotype and functionality (20, 23, 113–115). Higher frequencies of Vδ2 T cells have been found in elite controllers and the frequency of cervical Vδ2 T cells has been correlated with SIV viral load, pointing towards a beneficial, potentially immunomodulatory role of this subset (62, 63, 116).

**Figure 3:**
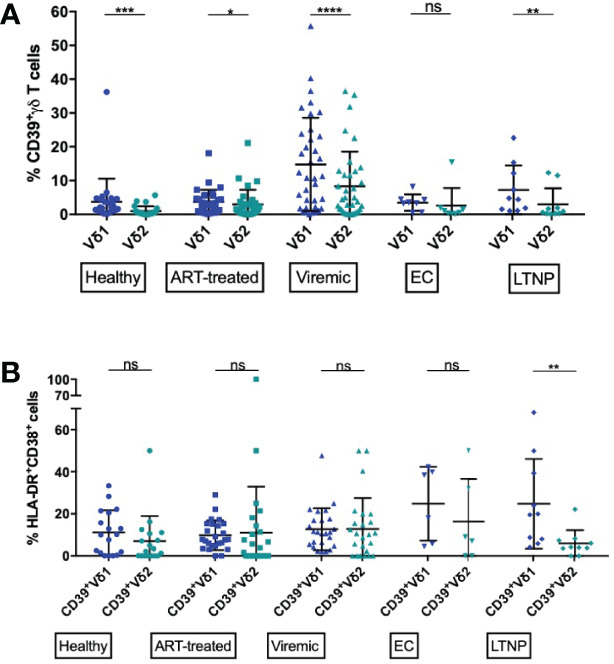
Frequency of CD39+ Vδ1 and Vδ2 cells among total and activated γδ T cells. **(A)** Frequency of CD39+ Vδ1 versus Vδ2 γδ T cells. **(B)** Frequency of activated (HLA-DR+CD38+) CD39+ Vδ1 versus Vδ2 γδ T cells. ns, non-significant p≥0,05; *p<0,05; **p≤0,01; ***p≤0,001; ****p≤0,0001.

We aimed to investigate the expression pattern of CD39+ Vδ1 versus CD39+ Vδ2 γδ T cells in healthy individuals and HIV patients, with the idea that CD39+ Vδ2 γδ T cells might have a stronger immunomodulatory function. In general, the expression of CD39 on the Vδ2 T-cell subset was significantly lower than on the Vδ1 γδ T-cell subset in all study groups except EC (**Figure 3A**). Also, there was a marked increase of CD39+ Vδ1 and Vδ2 γδ T cells in PBMC from viremic patients compared to healthy controls. The largest differences in CD39 expression between Vδ1 and Vδ2 γδ T cells were observed in healthy and viremic individuals (healthy: 3,7% vs. 1,0%, p=0,0001; viremic: 14,8% vs. 6,98,3%, p<0,0001). In samples from patients on ART, the expression levels of CD39+ γδ T cells were similar in the Vδ1 and Vδ2 subsets (3,7% vs. 3,0%, p=0,0266). The same was observed in PBMC from EC, where no statistically significant differences were detected between Vδ1 and Vδ2 γδ T cells (3,5% vs. 2,6%, p=0,3125). In samples from LTNP, the frequency of CD39+ cells was also significantly higher among Vδ1 compared to Vδ2 γδ T cells (7,2% vs. 3,0%, p=0,0039) (**Figure 3A**).

Overall, CD39 expression on total γδ T cells correlated with immune activation in HIV patients (**Supplementary Figure 5A**). We thus compared the frequency of activated (HLA-DR+CD38+) CD39+ Vδ1 and CD39+ Vδ2 subsets (**Figure 3B** **)**. We observed similar frequencies in all studied groups except LTNP, where the frequency of activated CD39+ Vδ2 was significantly lower than the frequency of activated CD39+ Vδ1 γδ T cells (CD39+ Vδ1 vs. CD39+ Vδ2: healthy: 11,2% vs. 7,0%; ART: 9,8% vs. 11,0%; viremic: 12,7% vs. 12,8%; EC: 24,8% vs. 16,4%; LTNP: 24,8% vs. 6,0%, p=0,0020) (**Figure 3B**). Interestingly, regardless of disease status, total Vδ1 γδ T were significantly more activated than Vδ2 γδ T cells (data not shown).

**Figure 4:**
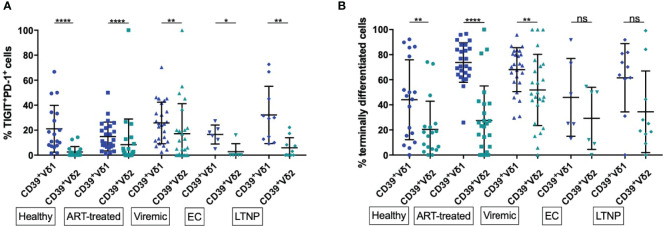
Frequency of exhausted and terminally differentiated cells among CD39+ Vδ1 versus CD39+ Vδ2. **(A)** Frequency of exhausted (TIGIT+PD-1+) CD39+ Vδ1 versus Vδ2 γδ T cells **(B)** Frequency of CD39+ Vδ1 versus Vδ2 γδ T cells at a late stage of differentiation (CD27-CD28-). *p<0,05; **p≤0,01; ****p≤0,0001.

In chronic, untreated HIV infections, an increase of terminally differentiated, exhausted, and dysfunctional CD8+ and CD4+ effector T cells has been described (82, 117, 118). We thus also assessed the differentiation and exhaustion status of Vδ1 and Vδ2 γδ T cells in conjunction with CD39 expression, taking the co-expression of the exhaustion markers PD-1 and TIGIT as an indicator of exhaustion and the absence of CD27 and CD28 as an indicator for a late stage of differentiation (**Figure 4**) (119).

We found significantly lower levels of exhausted (PD-1+TIGIT+) γδ T cells among CD39+ Vδ2 compared to CD39+ Vδ1 γδ T cells regardless of disease status (healthy: 21,15% vs 2,56%, p<0,0001; ART: 14,90% vs. 8,49%, p<0,0001; viremic: 25,76% vs. 17,22%, p=0,0018; EC: 16,5% vs. 2,67%, p=0,0312; LTNP: 32,21% vs. 5,87, p=0,0020; **Figure 4A**). As observed for activation, a significantly higher frequency of total Vδ1 compared to Vδ2 γδ T cells were exhausted (PD-1+ TIGIT+) in all study groups (data not shown).

We found a higher frequency of cells with late differentiation status (CD27-CD28-) among CD39+ Vδ1 compared to CD39+ Vδ2 γδ T cells in healthy individuals and HIV patients regardless of the disease status (**Figure 4B**). These differences of differentiation between CD39+ Vδ1 and Vδ2 γδ T cells were statistically significant except for the γδ T cells of EC and LTNP (healthy: 44,02% vs. 20,4%, p=0,0032; ART: 73,71% vs. 27,44%, p<0,0001; viremic: 68,08% vs. 51,86%, p=0,0018; EC: 45,97% vs. 29,24%, p=0,3125; LTNP: 61,56% vs. 34,42%, p=0,0645).

In summary, markers of T-cell exhaustion were more frequently expressed among CD39+ Vδ1 than CD39+ Vδ2 T cells regardless of disease status, and late differentiation of cells was more often present in CD39+ Vδ1 than CD39+ Vδ2 T cells of healthy individuals and HIV progressors.

**Figure 5:**
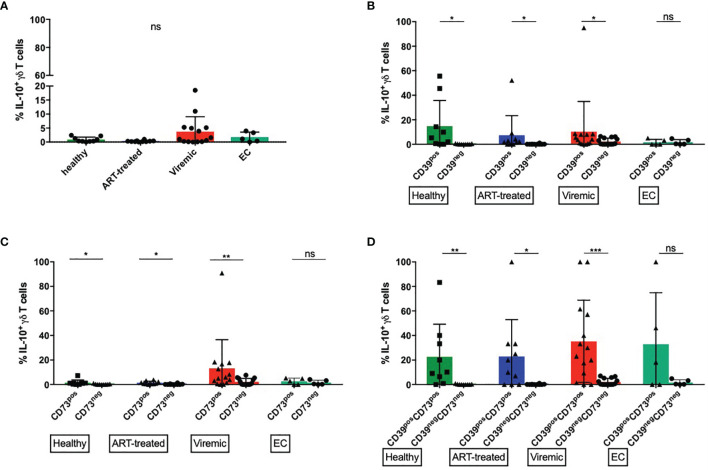
CD39+ γδ T cells produce more IL-10 upon *in vitro* stimulation than CD39- γδ T cells. **(A)** Frequency of IL-10 producing total γδ T cells **(B)** Frequency of IL-10 producing CD39+ versus CD39- γδ T cells. **(C)** Frequency of IL-10 producing CD73+ versus CD73- γδ T cells. **(D)** Frequency of IL-10 producing CD39+CD73+ versus CD39-CD73- γδ T cells. ns, non-significant p≥0,05; *p<0,05; **p≤0,01; ***p≤0,001.

While CD39 is upregulated during generalized immune activation in many lymphocyte populations, we and others could also define CD39 as a marker of several immunomodulatory populations in healthy individuals and HIV patients (78, 81, 98, 99, 104, 111). To characterize the functional profile of CD39+ γδ T cells and to assess their potential immunomodulatory effector functions, we performed intracellular cytokine stainings of γδ T cells for IL-10, IL-2, IFN-γ, TNF-α, TGF-β, and Granzyme-B after unspecific stimulation of PBMC with PMA and ionomycin (**Figure 5** and **Supplementary Figure 8**).

### Expression of CD39 and CD73 Marks γδ T Cells That Produce IL-10 at High Levels After *In Vitro* Stimulation

In untreated HIV infection, multiple cell types secrete IL-10 that suppresses virus-specific T cells and thereby inhibits virus clearance (100). In mice, a population of regulatory γδ T cells that secrete IL-10 have been described (99). However, there are few data on the IL-10 secretion of peripheral γδ T cells in HIV. Thus, we specifically examined the production of IL-10 by γδ T cells.

The frequencies of IL-10+ cells were generally lower among total γδ T cells than in CD39+ or CD39+CD73+ γδ T cells (**Figure 5A**). There were no statistically significant differences between the study groups. Interestingly, the highest frequency of IL-10+ γδ T cells was found in viremic HIV-infected patients, who also expressed the highest amount of CD39 (**Figure 1A**). We thus compared the frequency of IL-10 producing CD39+ and CD39- γδ T cells (**Figure 5B**). In samples from healthy donors, viremic HIV-infected individuals and patients on ART, the frequency of IL-10 producing γδ T cells was significantly higher among CD39+ than CD39- cells (healthy: 14,8% vs. 0,2%, p=0,0195; ART: 7,4% vs. 0,2%, p=0,0156; viremic: 10,4% vs. 2,3%, p=0,0171). In samples from EC, we detected similar frequencies of IL-10 producing γδ T cells between CD39+ and CD39- cells (1,7% vs. 1,7%, p=0,5556).

Similarly, in all groups but EC, the frequency of IL-10+ cells was significantly higher among CD73+ than CD73- γδ T cells upon *in vitro* stimulation (healthy: 1,5% vs. 0,3%, p=0,0273; ART: 1,4% vs. 0,2%, p=0,0273; viremic: 13,2% vs. 2,2%, p=0,0034), although the frequency of IL-10+ cells was overall lower than in CD39+ γδ T cells (**Figure 5C**).

We next assessed the capacity of the small population of CD39+CD73+ versus CD39-CD73- γδ T cells to produce IL-10 (**Figure 5D**). Interestingly, CD39+CD73+ γδ T cells produced more IL-10 than CD39-CD73- γδ T cells regardless of the disease status. In all groups but EC, the differences between CD39+CD73+ and CD39-CD73- γδ T cells reached statistical significance (healthy: 22,6% vs. 0,2%, p=0,0078; ART: 22,9% vs. 0,2%, p=0,0156; viremic: 35,2% vs. 2,2%, p=0,0005; EC: 32,8% vs. 1,7%, p=0,2500).

Lastly, we examined whether there were differences between the IL-10 production of CD39+ Vδ1 versus CD39+ Vδ2 γδ T cells (**Supplementary Figure 9**). The frequency of pooled (combined data from all study groups) IL-10+ CD39+ Vδ2 cells was higher than the frequency of pooled IL-10+ CD39+ Vδ1 γδ T cells (16,2% vs. 3,7%, p=0,01). Comparing the study groups, the frequency of IL-10+ CD39+ Vδ2 γδ T cells was highest in samples from healthy donors and decreased in samples from HIV-infected individuals (31,3% vs. ART: 10,5%; viremic: 15,0%; EC: 4,2%), but these differences did not reach statistical significance.

Taken together, we found that the ability of γδ T cells to produce IL-10 is higher among the CD39+ than the CD39- or the CD73+ subset. The highest frequency of IL-10 producing cells was found among CD39+CD73+ γδ T cells, a subset that is very scarce in PBMC. Also, the percentage of IL-10 producing cells tended to be higher in CD39+ Vδ2 compared to CD39+ Vδ1 γδ T cells and was declined in samples from patients with HIV compared to healthy individuals.

### Cytokine Profiles of CD39+ Versus CD39- Vδ2 γδ T Cells

**Figure 6:**
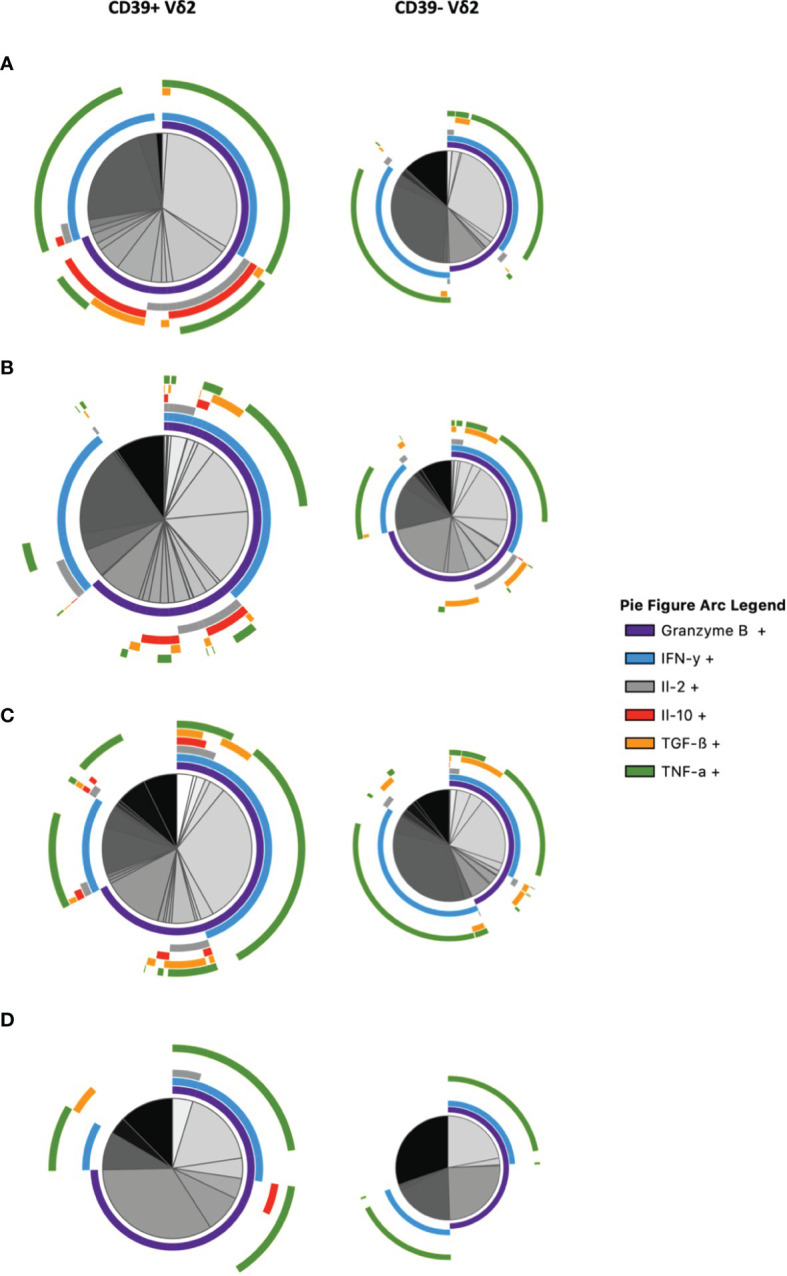
Multidimensional comparison of the cytokine profiles of CD39+/- Vδ2 γδ T cells *via* SPICE analysis. Samples from **(A)** healthy controls **(B)** HIV-infected viremic patients **(C)** HIV-infected patients on ART **(D)** elite controllers. Overlapping arcs denote cell populations that co-express the respective cytokines. See also **Supplementary Figure 10** for a detailed pie figure legend.

To get a better overview of their functional profile and polyfunctionality in terms of secretion of relevant cytokines, we conducted a multidimensional analysis of the cytokines secreted by CD39+ versus CD39- Vδ2 γδ T cells *via* SPICE analysis (**Figure 6** and **Supplementary Figure 10**). In addition to IL-10, we analyzed the expression of Granzyme B, IFN-γ, IL-2, TGF-ß, and TNF-α after *in vitro* stimulation. The analysis illustrates the differences between CD39+ and CD39- Vδ2 γδ T cells. There are several CD39+ Vδ2 γδ T-cell subpopulations in samples from healthy individuals that co-expressed the anti-inflammatory cytokines IL-10 and TGF-ß (illustrated as red and orange arcs). These subpopulations are found to a lesser extent in the corresponding CD39- Vδ2 γδ T cells but are also strongly reduced in samples from HIV-infected individuals. Also, pro-inflammatory cytokines such as IFN-γ or TNF-α (light blue and green arcs) are distributed differently between CD39+ and CD39- Vδ2 γδ T cells, indicating that these two subtypes might also differ functionally.

To summarize the polyfunctionality of CD39+ versus CD39- Vδ2 γδ T cells, we plotted the number of different cytokines that can be produced by the respective subset (**Supplementary Figure 11** **)**. In samples from healthy controls, all of the CD39+ Vδ2 γδ T cells expressed at least two cytokines, with approximately three-quarters of the CD39+ Vδ2 γδ T cells expressing three cytokines. By contrast, most cells in the respective CD39- subset did not produce any of the analyzed cytokines, and only approximately 15% produced three different cytokines. In samples from patients with HIV, the differences between CD39+ and CD39- Vδ2 γδ T cells were less pronounced. Vδ2 γδ T cells from viremic patients mostly produced none of the analyzed cytokines, regardless of CD39 expression. Similar proportions of the CD39+ and CD39- Vδ2 subsets produced three or fewer of the analyzed cytokines.

In patients on ART, about half of the CD39+ Vδ2 γδ T cells produced none of the analyzed cytokines; however, a considerable fraction produced three different cytokines and two smaller fractions produced one or two cytokines. CD39- Vδ2 T cells of patients on ART had a similar pattern to CD39- Vδ2 γδ T cells from viremic individuals. Interestingly, a small fraction in all subsets of viremic and individuals on ART produced four different cytokines. Finally, the majority of CD39+ Vδ2 γδ T cells from EC expressed one cytokine, followed by one-third that produced three different cytokines and a smaller fraction that produced two different ones. CD39- Vδ2 γδ T cells from EC mostly did not produce any of the analyzed cytokines, a small fraction produced three different ones, followed by minor fractions producing two or one cytokines.

Taken together, we found diverging cytokine profiles of CD39+ and CD39- Vδ2 γδ T cells. In HIV infection, γδ T cells lost their polyfunctionality in parts and produced fewer anti-inflammatory cytokines.

### Moderate Changes in the Composition, but Divergent Cytokine Repertoire of γδ T Cells From HIV Elite Controllers

We analyzed samples from HIV elite controllers, where we found striking disparities in the phenotype and functionality of γδ T cells compared to samples from infected individuals who had a high detectable load (viremic) or were on antiretroviral medication (ART). First, the frequency of CD39+ γδ T cells was not elevated compared to samples from healthy controls (**Figure 1**), and there were less pronounced changes in the Vδ1/Vδ2 ratio (**Supplementary Figure 1**). Also, the frequency of CD73+ γδ T cells was not significantly decreased compared to healthy controls (**Figure 1**). When comparing the features between Vδ1 and Vδ2 γδ T cells, we did not find differences between the frequencies of CD39+ (**Figure 3**) or CD73+ (data not shown) Vδ1 and Vδ2 γδ T cells in samples from EC.

Interestingly, only low frequencies of IL-10 producing CD39+ γδ could be detected compared to HIV progressors (**Figure 5**), and CD39+/- Vδ2 γδ T cells from EC produced a lower number of different cytokines than γδ T cells from healthy controls (**Supplementary Figure 11**).

Long-term non-progressors maintain stable CD4+ T-cell counts despite low to intermediate plasma viremia. The differences between LTNP and HIV progressors were not as pronounced as between EC and the latter (105, 106, 120). By contrast, we observed similar changes in the expression pattern of CD39 and CD73 and the phenotype of CD39+/CD73+ γδ T cells compared to patients on ART and viremic individuals.

## Discussion

γδ T cells are part of the first-line defense against pathogens since they exert direct cytotoxic functions independent of MHC proteins (15, 17), but also immunomodulatory γδ T cells have been described in different immunological settings such as cancer and inflammatory bowel disease (121, 122). Alterations of CD39 and CD73 expression on several lymphocyte populations have been described and are important for HIV pathogenesis (82, 88, 90, 101, 123). In the current study, a detailed phenotypical and functional characterization of CD39 and CD73 expression on different γδ T-cell populations from healthy individuals and HIV patients with different disease courses was carried out.

Our results show that the expression pattern of these ectoenzymes is associated with distinct functional states and can be used as a marker to identify activated cells. We find significant differences in CD39 and CD73 expression on total γδ T cells, as well as on Vδ1 and Vδ2 cells between healthy and HIV-infected individuals depending on the clinical status. Importantly, we define a small population of γδ T cells co-expressing CD39 and CD73 that produce IL-10 after *in vitro* stimulation in healthy individuals and HIV patients.

In chronic HIV infection, IL-10 concentrations in the blood plasma were reported to increase over time, mediated by different lymphocyte populations (100, 124). The level of IL-10 production correlates with disease progression and causes reversible T-cell dysfunction to enable a balance between protective responses and immunopathology. IL-10 expression is associated with the expression of CD39 and the frequency of CD39+ cells secreting IL-10 has been correlated with viral load and immune activation (99, 101–103). Furthermore, IL-2 production is inhibited *via* the CD39/ADO pathway in CD39+ Tregs (125, 126).

We and others demonstrate that CD39, PD-1, and IL-10 were increased on γδ T cells in viremic HIV infection and provide an immunosuppressive environment in which the immune system is unable to clear the HI virus. Interestingly, our data show that neither IL-10 nor PD-1 nor CD39 increase strongly in EC, who can control the infection spontaneously.

One way to (partially) revert the function of virus-specific effector T cells (e.g. Vδ1 cells) is by combined checkpoint inhibitor blockade and blockade of adenosine signaling. Interestingly, CD39+ T cells CD8+ often also express PD-1 and other markers of cellular exhaustion. Li et al. demonstrated a reversion of CD8 exhaustion by concomitant blockade of PD-1 and adenosine pathways (127).

(IL-10+CD39+) γδ T cells could be reactivated by blockade of IL-10 or PD-1, CD39, or combinations thereof (80). Restored CD4 T cell function was previously achieved by immune checkpoint blockade of PD-1 and IL-10 in HIV-infected patients and Tang et al. demonstrated an improved function of MAIT cells during HIV/tuberculosis infection (128,129).

Overall, a strong correlation between the frequency of CD39+ and CD73+ γδ T cells and immune activation as well as disease progression (viral load and CD4+ T-cell count) could be determined. Thus, we propose that CD39 expression as well as the down-regulation of CD73 on γδ T cells can be seen as markers of activation, which has also been proposed in other immunological and disease contexts (99, 112, 130). The shifts of CD39 and CD73 have important implications regarding homing, functionality, nucleotide metabolism (that can occur in cis and/or trans), as well as interaction with other lymphocyte populations (131).

In this study, only HIV elite controllers showed an expression of both enzymes on γδ T cells comparable to healthy controls, while the expression pattern of CD39 and CD73 was altered in viremic and not fully normalized in individuals on ART. Viremic HIV patients had the highest CD39 expression on their γδ T cells – and these CD39+ γδ T cells produced the most IL-10 after *in vitro* stimulation. CD39 has lately been defined as a potential marker of immunomodulatory cells like Treg and NK cells, and CD39+ Vδ2 T cells might have a peculiar immunomodulatory role in HIV infection (64, 101, 104). One might interpret the CD39+CD73+IL-10+ γδ T cells as a counter-reaction to viremia to abrogate excessive inflammation, and speculate how this IL-10 production can inhibit HIV-specific immune responses and therefore act detrimentally in HIV pathogenesis (100, 124). Our data from elite controllers, who maintain low levels of CD39 and produce considerably less IL-10 than viremic HIV-infected patients, fit this hypothesis.

To see whether the observed alterations were specific for HIV infection, we also examined samples from patients with other viral infections, i.e. HCV or HBV. There, no significant increase of CD39+ γδ T cells compared to healthy controls could be measured. Similar to HIV infection, there is a loss of peripheral Vδ2 cells, an expansion of peripheral Vδ1 cells, and strong immune activation in (chronically) HBV‐infected subjects (132, 133). The transcriptional pathways and factors (e.g. cytokines) that regulate CD39 and CD73 expression need to be better defined for γδ T cells (see also below), but also other lymphocyte populations. Several cytokines that have also been shown to be altered in HIV infection regulate CD39 on lymphocytes (134). For example, IL-6 and TGF-ß are likely to lead to an up-regulation of CD39 on lymphocytes (134). *In vitro*, TCR engagement and IL-2 increased CD39 expression. In mice, IL-27 signaling triggers CD39 expression in Tregs by a STAT-1-dependent mechanism (134). Another factor that influences CD39 expression on human T cells includes genetic variations (single nucleotide polymorphisms) (134).

Overall, the role of γδ T cells in HIV remains elusive and seems double-edged (41). Pan Vδ2 γδ T cells have been associated with a protective role in HIV: a study in non-human primates identified a relationship between cervical Vδ2 frequency and SIV viral load, and Vδ2 γδ T cells expressing CD16 are capable of mediating potent ADCC (62,135). Another study reported that elite controllers maintain significantly higher frequencies of Vδ2 T cells than untreated patients or those on ART (63, 64). We also find a higher frequency of Vδ2 γδ T cells in samples from long-term non-progressors compared to HIV progressors.

On the other hand, Soriano-Sarabia et al. report that replication-competent HIV could be recovered from purified Vδ2 γδ T cells in 14 of 18 long-term ART recipients and thus concluded that these cells form part of the viral reservoir (55). It has been demonstrated that Vδ2 cells express high levels of the HIV co-receptors CCR5 and α4β7 which contribute to their infectibility (53–56). CD39+ cells are more activated than CD39- cells, thereby inducing transient expression of CD4 on Vδ2 γδ T cells *in vivo* and promoting infection. Follow-up studies should test the hypothesis that CD39+ Vδ2 γδ T cells form part of the viral reservoir, as has been shown for CD39+ Tregs (55, 79). In naïve Tregs, a correlation between HIV DNA and frequency of CD39+ naïve Tregs was demonstrated by Song et al. (79)

We observed a loss of polyfunctionality, defined as cells capable of producing three or more cytokines after *in vitro* stimulation, within the CD39+ Vδ2 γδ T-cell population of viremic HIV patients that was not fully regained in γδ T cells of HIV-infected individuals under ART. These results are in line with results from Casetti et al., who also measured a reduction of polyfunctionality (cytokine/chemokine production and cytotoxicity) in Vδ2 γδ T cells from ART-treated patients (136, 137).

This first study on the CD39 and CD73 expression pattern and functionality of γδ cells in HIV patients has some limitations. A first one is given by the limited number of parameters that could be measured in a respective panel by flow cytometry analysis. In future studies, the expression of CD16, CD56, and NKG2D, as well as the transcriptional profile [FOXP3, HIF-1, and AhR (138–141)], should be included in the flow cytometry experiments or assessed, e.g. by use of single-cell transcriptional RNA expression analysis (142). NKG2D can activate γδ T cells in an innate TCR-independent manner and is expressed by the vast majority of Vδ2 T cells (29, 43, 143, 144).

We found that the frequency of circulating γδ T cells expressing both CD39 and CD73 is particularly low. However, CD39+CD73+ γδ T cells had the highest frequencies of IL-10-producing cells after *in vitro* stimulation. One could hypothesize that this small sub-population of CD39+CD73+ γδ T cells that secretes an anti-inflammatory cytokine stays relatively unaffected from HIV infection. By contrast, the majority of γδ T cells show shifts of the CD39/CD73 expression ratio comparable to the changes observed in the effector cell compartment, most likely due to generalized immune activation in HIV (86, 145). It will be interesting to further investigate this scarce population of cells alongside the other γδ T-cell populations, especially regarding their suppressive capacities. Since the frequency of peripheral CD39+, CD73+ γδ T cells is too low for live cell sorting and subsequent co-culture with activated T cells, transcriptional analyses such as single-cell sequencing with regard to the transcriptome will have to be used to understand the capabilities of this and other γδ T-cell populations. Alternatively, γδ T-cell subpopulations could be expanded *in vitro* before life-sorting, co-culture, and flow-based read-out, with the disadvantage that this expansion may alter the phenotype and the function of the γδ T cells.

Previous studies point towards a regulatory role of CD39 in the inflammatory microenvironment of the gut which is caused i.a. by microbial ATP (109). Our group has previously demonstrated that the frequency of mucosa-derived CD39+ γδ T cells is decreased in patients with inflammatory bowel diseases compared to healthy controls (81). Upon stimulation, these cells produced less IL-17 and more IL-10 than CD39- γδ T cells, also pointing towards a regulatory phenotype of these cells. At the same time, the number of Tregs in the mucosal compartment was increased, which could serve as a compensatory mechanism for the loss of the CD39+ γδ T cells (81). The gut is one of the major sites for virus dissemination and formation of the viral reservoir (146–148). It will therefore be worthwhile to examine alterations in the number and function of CD39+ γδ T cells in the gut-associated lymphoid tissue (GALT) from HIV-infected individuals compared to healthy controls.

It will also be interesting to study patients with primary HIV infection. Bhatnagar et al. have demonstrated that Vδ2 γδ T cells transform from an anti-inflammatory phenotype in primary infection into a pro-inflammatory cytokine profile in chronic infection (35).

Taken together, the CD39/CD73 expression ratio on γδ T cells in untreated HIV is inversed and is associated with immune activation and disease progression. We find altered functionality and higher levels of IL-10 production in viremic HIV patients. Also, we defined a small population of CD39+CD73+ γδ T cells producing IL-10 at high frequencies after *in vitro* stimulation.

We hypothesize an immunomodulatory role of CD39+ and CD73+ γδ T cells in the pathogenesis of chronic HIV infection potentially mediated by IL-10 secretion. Similar to the deleterious role of suppressive cells in the microenvironment of tumors, the frequency of CD39+ γδ T cells was correlated with HIV disease progression in this study. This is further supported by our findings in elite controllers, who maintain stable frequencies of (IL-10-producing) CD39+ and CD73+ γδ T cells compared to healthy controls. Also, double-positive CD39+CD73+ produced significantly more IL-10 than γδ T cells expressing only one ectonucleotidase. Finally, a link between CD39 and IL-10 expression and disease progression has already been established in NK cells (90).

Future studies have to understand the role of adenine metabolism for γδ T cell function and elucidate the effects of the alterations of CD39 and CD73 expression on γδ T cells in HIV in more detail.

## Data Availability Statement

The raw data supporting the conclusions of this article will be made available by the authors, without undue reservation.

## Ethics Statement

The studies involving human participants were reviewed and approved by Institutional Review Board, Ärztekammer Hamburg, Hamburg, Germany. The patients/participants provided their written informed consent to participate in this study.

## Author Contributions

KK and MW have contributed equally to this work and share the first authorship. KK and JS designed the initial study design. MW and JS wrote the first draft of the manuscript together with KK. JS gave funding and was in charge of the overall research project. KK and MW conducted most of the experiments. A-DH, OD, H-JS, and JS recruited the patients and collected patient data. KK and MW analyzed the data under the supervision of JS, PH, and FH. MW and KK prepared the figures and got input from FH, JS and all other authors. PH and FH aided in interpreting the results. All authors discussed the results and critically reviewed the manuscript. All authors contributed to the article and approved the submitted version.

## Funding

FH, and JS get funding from the DFG (SFB1328 A12), JS and MW get funding by the H.W. & J. Hector Stiftung (project M2101). KK, MW, and JS get funding from the DZIF (TTU 04.816), JS and CA are also funded by the European Union Horizon 2020 program (European HIV Vaccine Alliance 681032).

## Conflict of Interest

The authors declare that the research was conducted in the absence of any commercial or financial relationships that could be construed as a potential conflict of interest.

## Publisher’s Note

All claims expressed in this article are solely those of the authors and do not necessarily represent those of their affiliated organizations, or those of the publisher, the editors and the reviewers. Any product that may be evaluated in this article, or claim that may be made by its manufacturer, is not guaranteed or endorsed by the publisher.

## Supplementary Material

Supplementary Figure 1 Stable frequencies of total γδ T cells (left) and inverted ratio (right) between Vδ1 and Vδ2 T cells in HIV infection.

Supplementary Figure 2 Gating Strategy to define total/pan γδ T cells and Vδ2 γδ T cells. First, dead cells, B cells, and monocytes were excluded. Then, single cells and lymphocytes were selected. After gating for CD3+ T cells, pan γδ T cells were defined. From there, Vδ2 γδ T cells were gated. Vδ1 γδ T cells were defined as Vδ2 negative. Exemplary plots from a long-term non-progressor are shown.

Supplementary Figure 3 Exemplary dot plots of γδ T cells expressing CD39 (upper panel) and CD73 (lower panel). Data are shown from selected healthy volunteers, HIV patients on ART, HIV-infected viremic individuals, elite controllers (EC), and long-term non-progressors (LTNP).

Supplementary Figure 4 Changes in the frequency of CD39+ and CD73+ γδ T cells in different viral infections. Left: Frequency of CD39+ γδ T cells. Right: Frequency of CD73+ γδ T cells in samples from healthy and viremic HIV-infected individuals, patients with acute and chronic HBV and patients with chronic HCV.

Supplementary Figure 5 Correlation between immune activation and frequency of CD39+ γδ T cells. The frequency of CD39+ γδ T cells correlates positively in HIV-infected patients **(A)** and inversely with immune activation in healthy individuals **(B)**. **(C)** Frequency of HLA-DR+CD38+ γδ T cells is higher among CD39+ than CD39- cells of regardless disease status. Graph **A** contains pooled data from HIV-infected patients including viremic and individuals on ART, EC, and LTNP.

Supplementary Figure 6 Correlation between frequency of activated γδ T cells and frequency of CD39+ γδ T cells. The frequency of activated (HLA-DR+CD38+) CD39+ γδ T cells correlates positively in HIV-infected patients (left) and inversely within healthy individuals (right). The left graph contains pooled data from HIV-infected patients including viremic and individuals on ART, EC, and LTNP.

Supplementary Figure 7 Kinetics of CD39 frequency after *in vitro* stimulation. Data from *in vitro* stimulated PBMC from healthy individuals.

Supplementary Figure 8 Exemplary dot plots of CD39+/CD39- Vδ1/Vδ2 γδ T cells expressing IL-10. Data are shown from viremic HIV-infected patients.

Supplementary Figure 9 Frequency of IL-10 producing total Vδ1 versus total Vδ2 and CD39+ Vδ2 γδ T cells. Data from pooled study subjects (healthy and HIV-infected individuals) and stratified according to disease status.

Supplementary Figure 10 Category legend to Figure 6. Plus denotes the existence of cells expressing the respective cytokine, minus the absence.

Supplementary Figure 11 Comparison of the degree of the polyfunctionality of CD39+ and CD39- Vδ2 γδ T cells *via* SPICE analysis. “Number of function” denotes the average number of cytokines produced by the respective subset. Samples from **(A)** healthy controls **(B)** HIV-infected viremic patients **(C)** HIV-infected patients on ART **(D)** elite controllers. Data is presented on a relative scale with median values.

Supplementary Table 1 Basic demographic and virologic data of the HBV- and HCV-infected patients (average, min - max).

Supplementary Table 2 Overview of fluorochrome-conjugated antibodies used for phenotypic characterization *via* flow cytometry (surface staining).

Supplementary Table 3 Overview of fluorochrome-conjugated antibodies used for functional characterization *via* flow cytometry (intracellular cytokine staining).
