# Supplementary material for: Inversed Ratio of CD39/CD73 Expression on γδ T Cells in HIV Versus Healthy Controls Correlates With Immune Activation and Disease Progression
Source: Front Immunol. 2022 Apr 22;13:867167. doi: 10.3389/fimmu.2022.867167 (PMC9074873; doi:10.3389/fimmu.2022.867167)
Supplement: Supplementary Figure 1 — Stable frequencies of total γδ T cells (left) and inverted ratio (right) between Vδ1 and Vδ2 T cells in HIV infection. [file DataSheet_1.pdf]

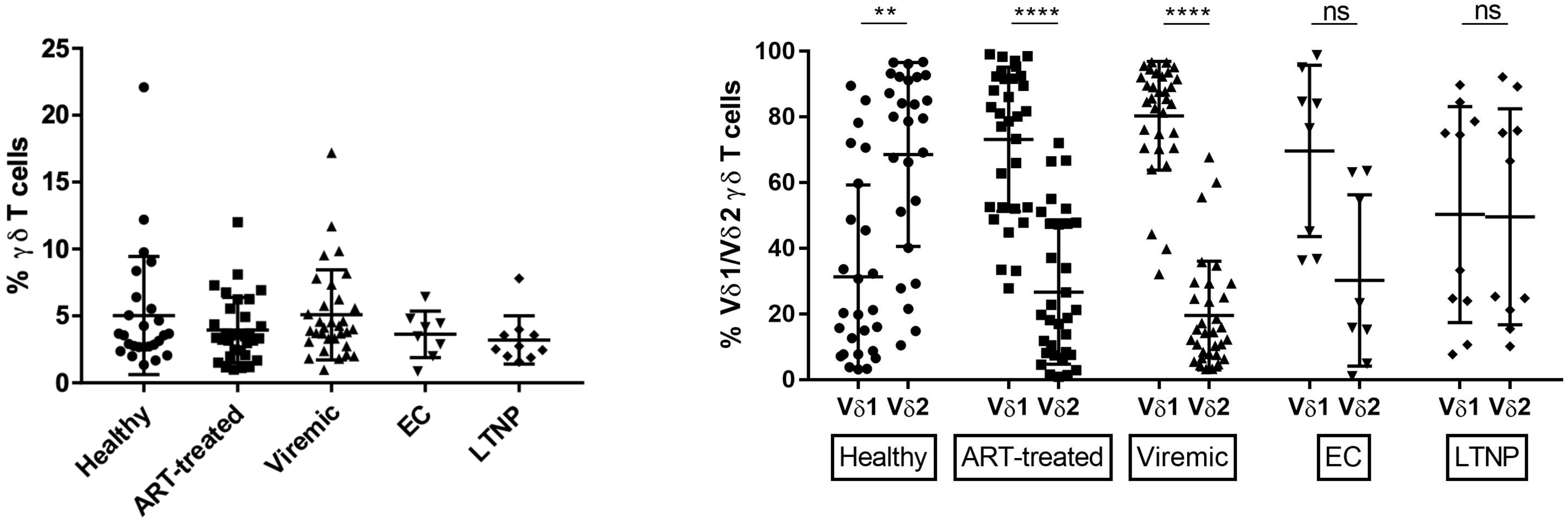

% γδ T cells
25
20
15
10
5
0
Healthy
ART-treated
Viremic
EC
LTNP
% Vδ1/Vδ2 γδ T cells
100
80
60
40
20
0
**
****
****
ns
ns
Vδ1 Vδ2
Vδ1 Vδ2
Vδ1 Vδ2
Vδ1 Vδ2
Vδ1 Vδ2
Healthy
ART-treated
Viremic
EC
LTNP

Suppl. Fig. 1

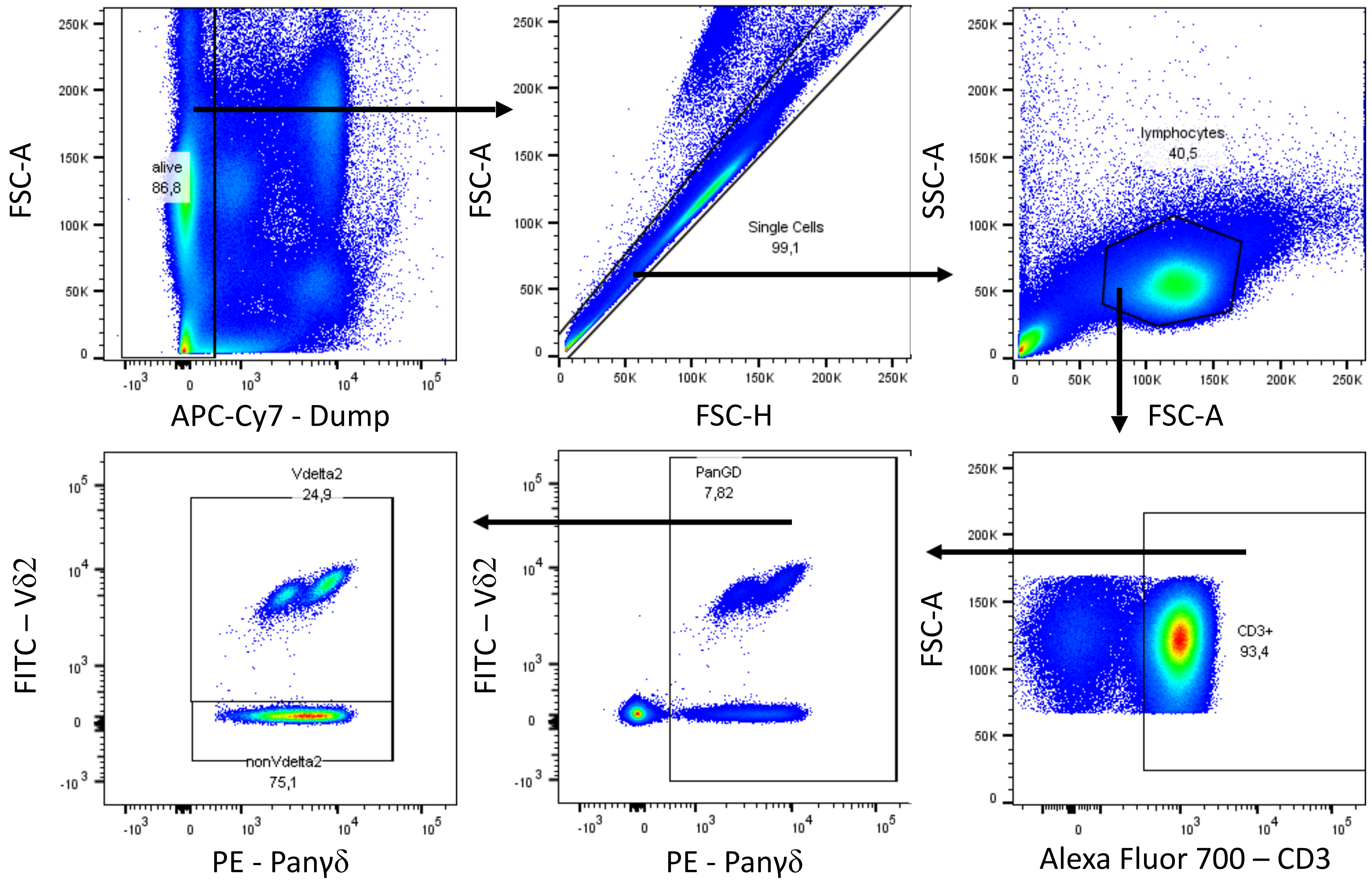
FSC-A
APC-Cy7 - Dump
alive
86,8
FSC-A
FSC-H
Single Cells
99,1
SSC-A
FSC-A
lymphocytes
40,5
FSC-A
Alexa Fluor 700 – CD3
CD3+
93,4
FITC – Vδ2
PE - Panγδ
PanGD
7,82
FITC – Vδ2
PE - Panγδ
Vdelta2
24,9
nonVdelta2
75,1

Suppl. Fig. 2

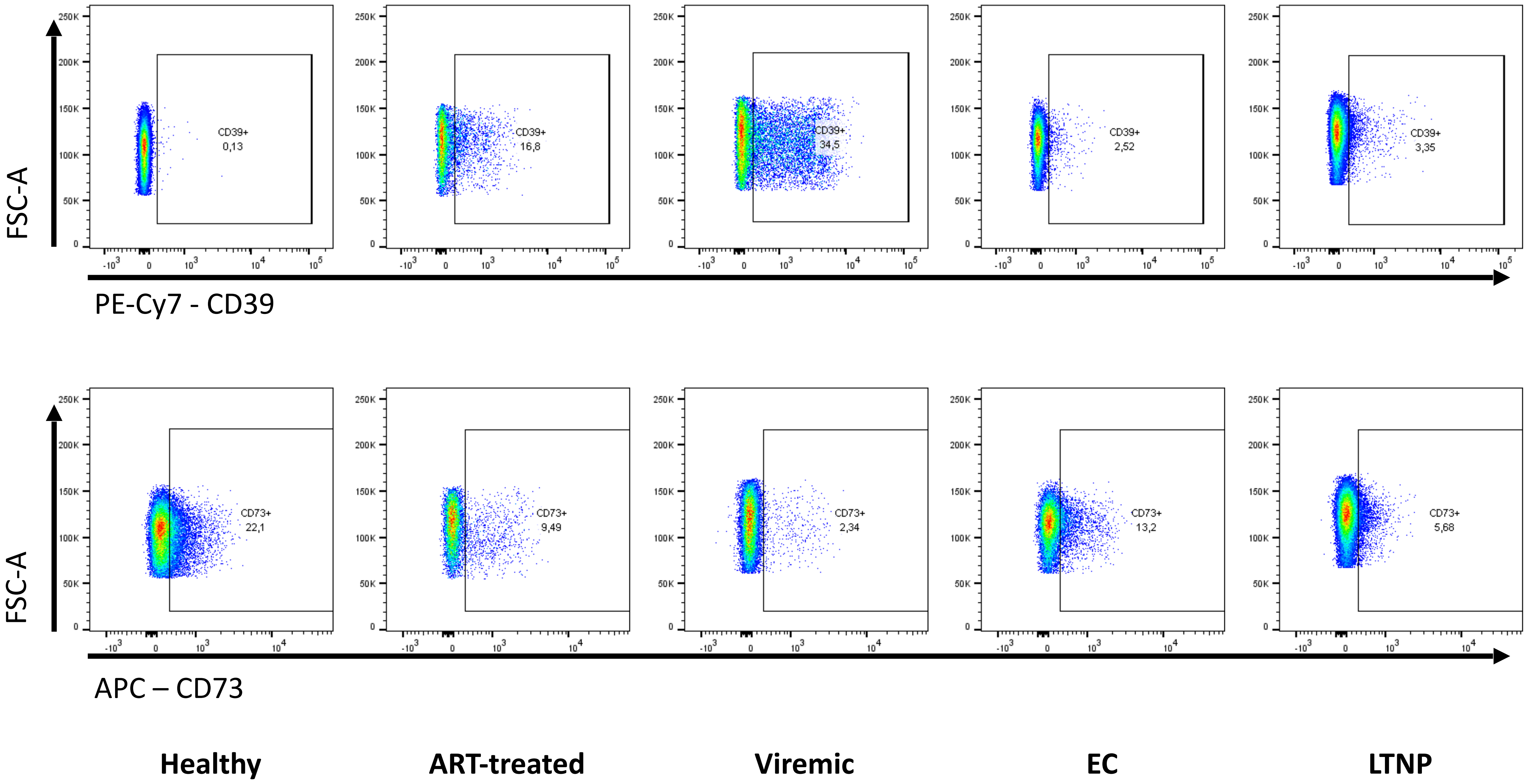
FSC-A
PE-Cy7 - CD39
CD39+ 0,13
CD39+ 16,8
CD39+ 34,5
CD39+ 2,52
CD39+ 3,35
APC – CD73
CD73+ 22,1
CD73+ 9,49
CD73+ 2,34
CD73+ 13,2
CD73+ 5,68
Healthy
ART-treated
Viremic
EC
LTNP

Suppl. Fig. 3

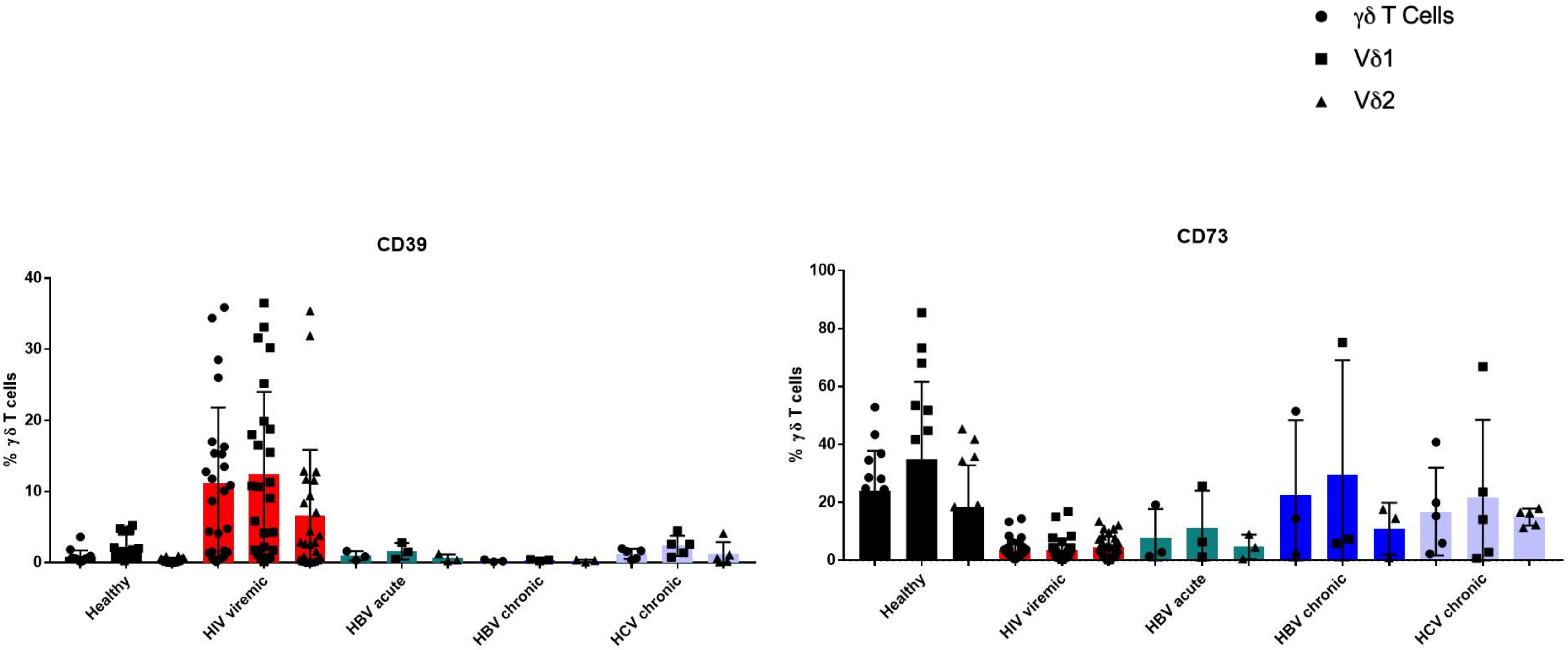
γδ T Cells
Vδ1
Vδ2
CD39
% γδ T cells
0
10
20
30
40
Healthy
HIV viremic
HBV acute
HBV chronic
HCV chronic
CD73
% γδ T cells
0
20
40
60
80
100
Healthy
HIV viremic
HBV acute
HBV chronic
HCV chronic

Suppl. Fig. 4

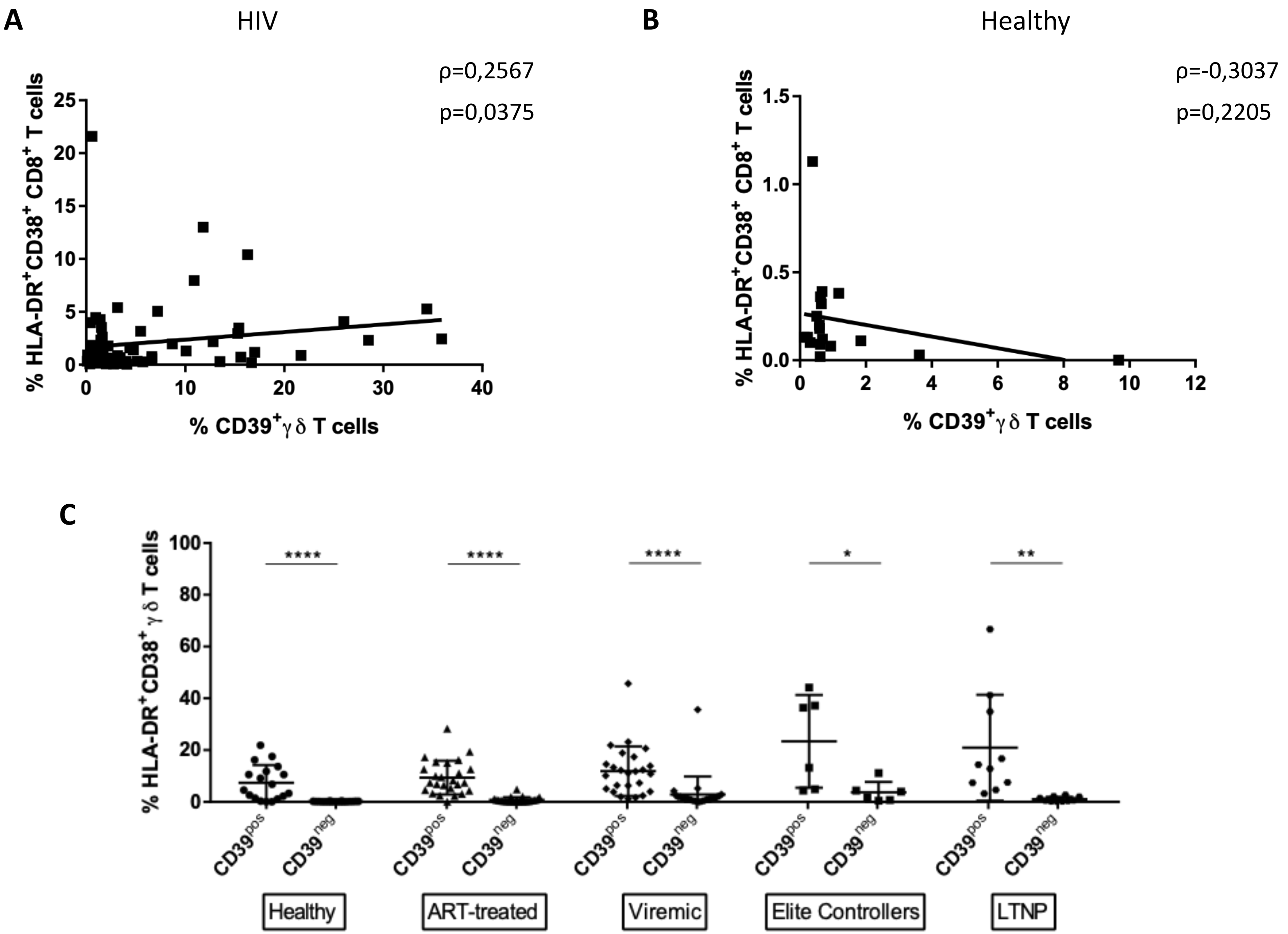
A
HIV
ρ=0,2567
p=0,0375
% HLA-DR+CD38+ CD8+ T cells
% CD39+γδ T cells
B
Healthy
ρ=-0,3037
p=0,2205
% HLA-DR+CD38+ CD8+ T cells
% CD39+γδ T cells
C
% HLA-DR+CD38+ γδ T cells
****
****
****
*
**
CD39pos
CD39neg
Healthy
ART-treated
Viremic
Elite Controllers
LTNP

Suppl. Fig. 5

HIV

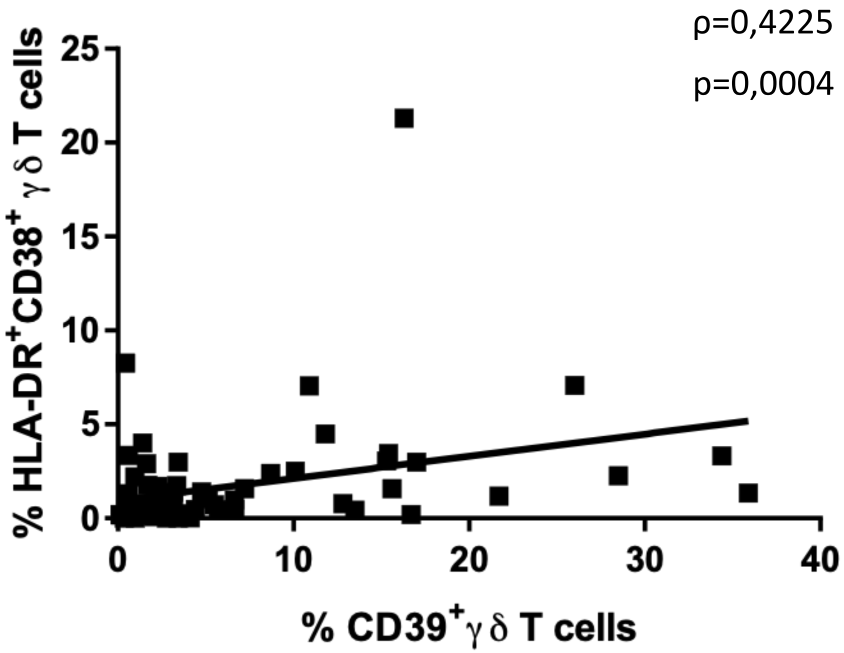

Healthy

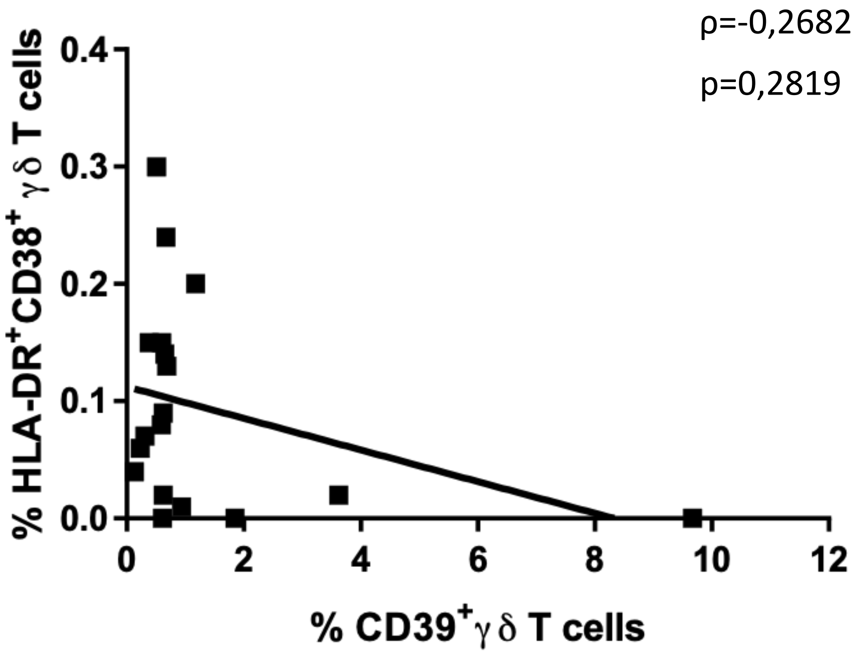

Suppl. Fig. 6

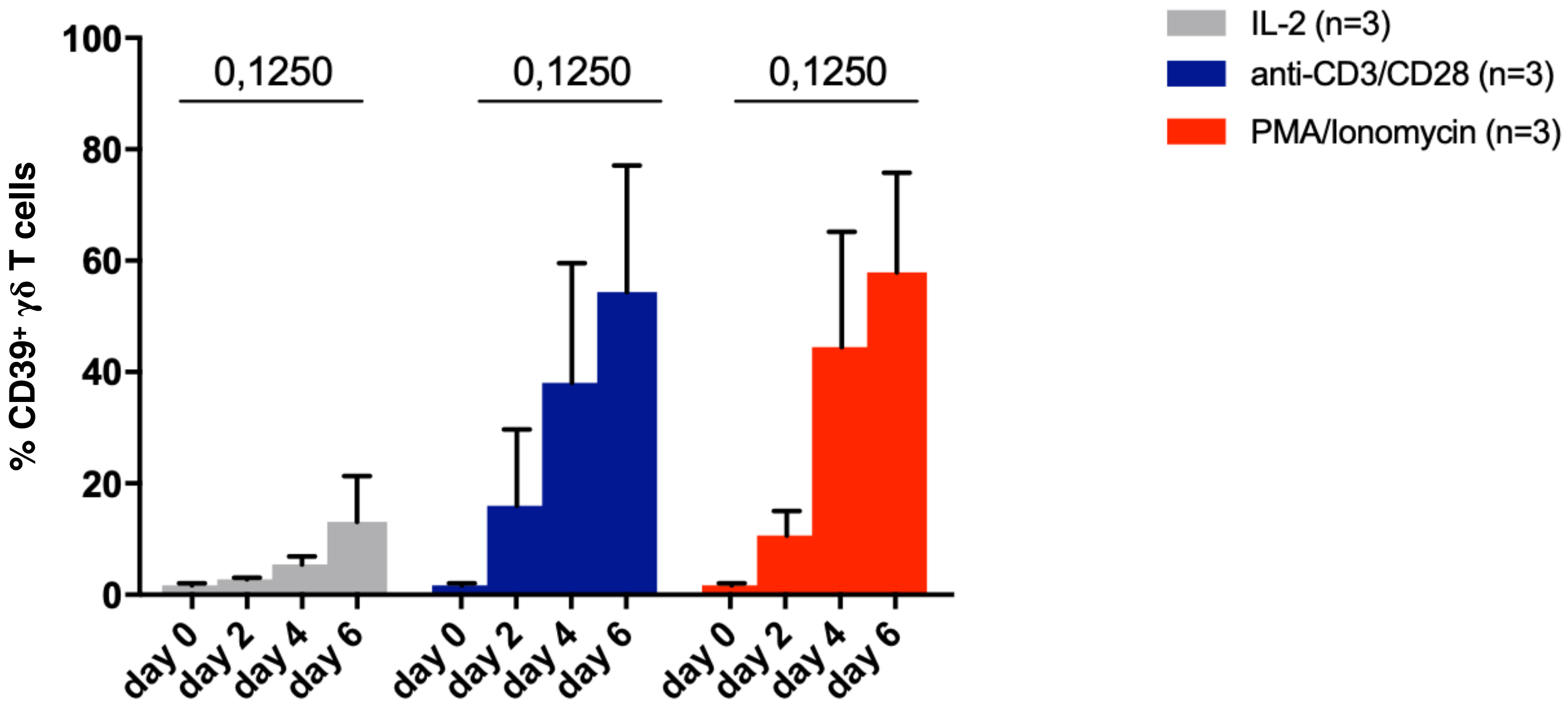

100
80
60
40
20
0
% CD39+ γδ T cells
0,1250
0,1250
0,1250
day 0
day 2
day 4
day 6
day 0
day 2
day 4
day 6
day 0
day 2
day 4
day 6
IL-2 (n=3)
anti-CD3/CD28 (n=3)
PMA/Ionomycin (n=3)

Suppl. Fig. 7

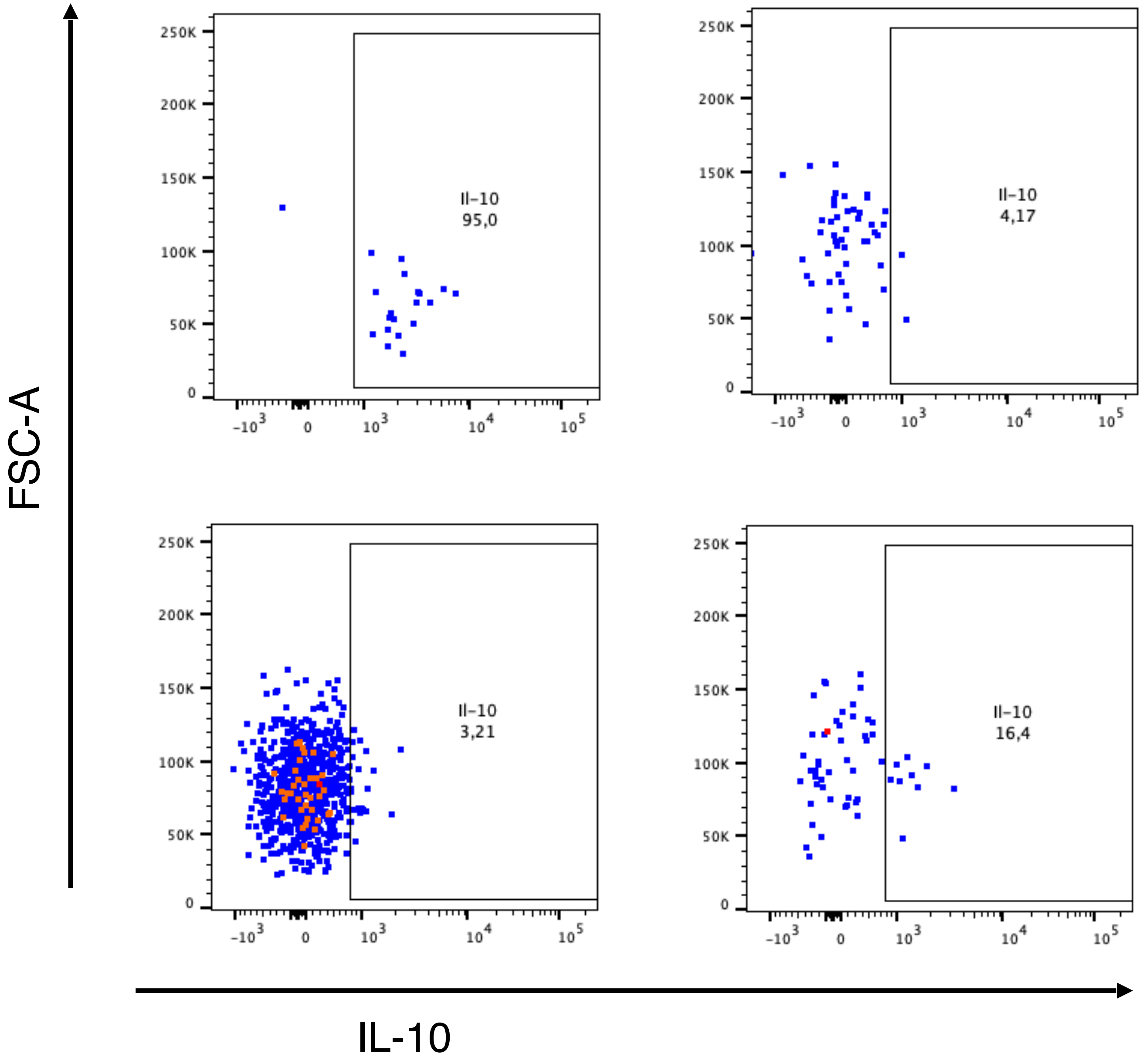

CD39+γδT	CD39-γδT
CD39+Vδ1	CD39+Vδ2

Suppl. Fig. 8

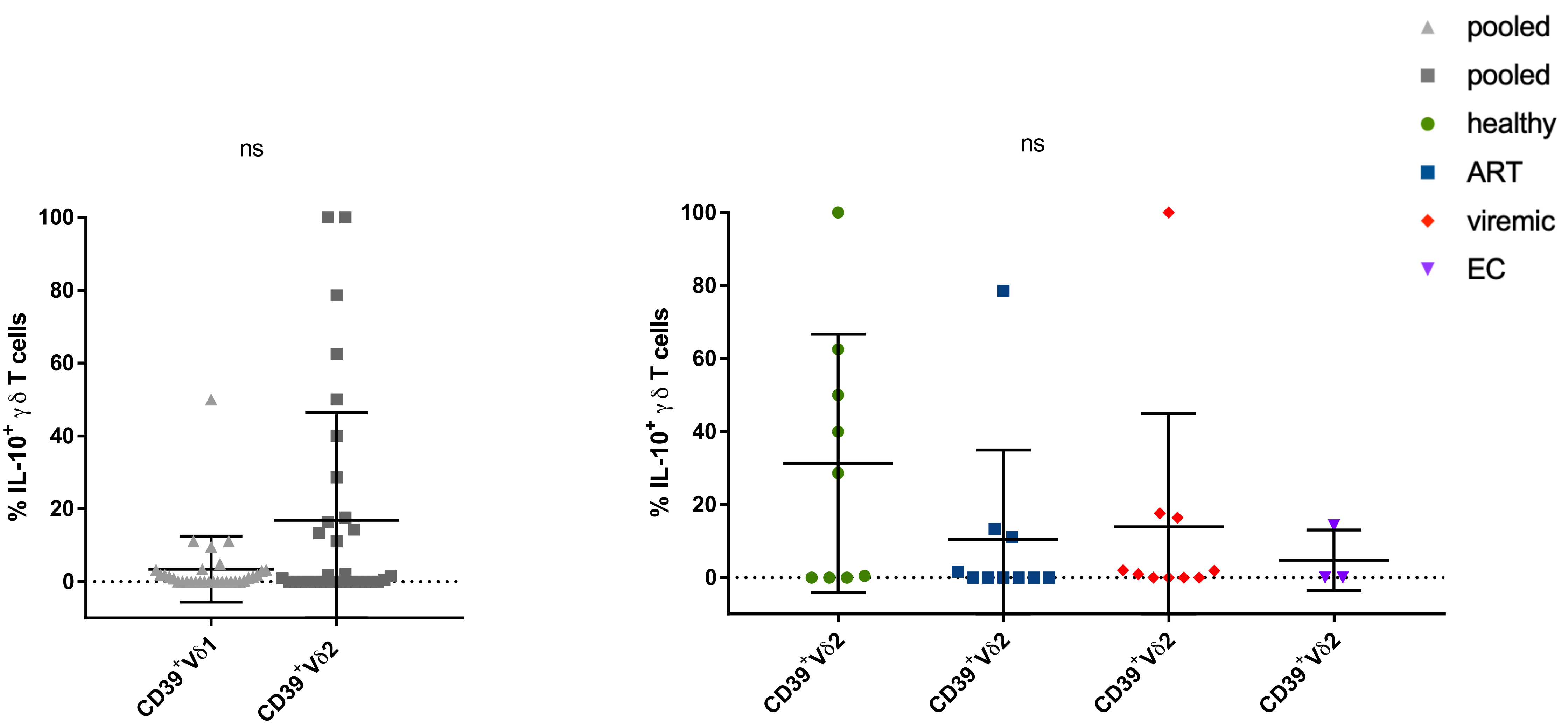
ns
100
80
60
40
20
0
% IL-10+ γδ T cells
CD39+Vδ1
CD39+Vδ2
ns
100
80
60
40
20
0
% IL-10+ γδ T cells
CD39+Vδ2
CD39+Vδ2
CD39+Vδ2
CD39+Vδ2
pooled
pooled
healthy
ART
viremic
EC

Suppl. Fig. 9

Categories

#	Granzyme B	IFN-y	Il-2	Il-10	TGF-ß	TNF-a
1	+	+	+	+	+	+
2	+	+	+	+	+	-
3	+	+	+	+	-	+
4	+	+	+	+	-	-
5	+	+	+	-	+	+
6	+	+	+	-	+	-
7	+	+	+	-	-	+
8	+	+	+	-	-	-
9	+	+	-	+	+	+
10	+	+	-	+	+	-
11	+	+	-	+	-	+
12	+	+	-	+	-	-
13	+	+	-	-	+	+
14	+	+	-	-	+	-
15	+	+	-	-	-	+
16	+	+	-	-	-	-
17	+	-	+	+	+	+
18	+	-	+	+	+	-
19	+	-	+	+	-	+
20	+	-	+	+	-	-
21	+	-	+	-	+	+
22	+	-	+	-	+	-
23	+	-	+	-	-	+
24	+	-	+	-	-	-
25	+	-	-	+	+	+
26	+	-	-	+	+	-
27	+	-	-	+	-	+
28	+	-	-	+	-	-
29	+	-	-	-	+	+
30	+	-	-	-	+	-
31	+	-	-	-	-	+
32	+	-	-	-	-	-

Categories

#	Granzyme B	IFN-y	Il-2	Il-10	TGF-ß	TNF-a
33	-	+	+	+	+	+
34	-	+	+	+	+	-
35	-	+	+	+	-	+
36	-	+	+	+	-	-
37	-	+	+	-	+	+
38	-	+	+	-	+	-
39	-	+	+	-	-	+
40	-	+	+	-	-	-
41	-	+	-	+	+	+
42	-	+	-	+	+	-
43	-	+	-	+	-	+
44	-	+	-	+	-	-
45	-	+	-	-	+	+
46	-	+	-	-	+	-
47	-	+	-	-	-	+
48	-	+	-	-	-	-
49	-	-	+	+	+	+
50	-	-	+	+	+	-
51	-	-	+	+	-	+
52	-	-	+	+	-	-
53	-	-	+	-	+	+
54	-	-	+	-	+	-
55	-	-	+	-	-	+
56	-	-	+	-	-	-
57	-	-	-	+	+	+
58	-	-	-	+	+	-
59	-	-	-	+	-	+
60	-	-	-	+	-	-
61	-	-	-	-	+	+
62	-	-	-	-	+	-
63	-	-	-	-	-	+
64	-	-	-	-	-	-

Suppl. Fig. 10

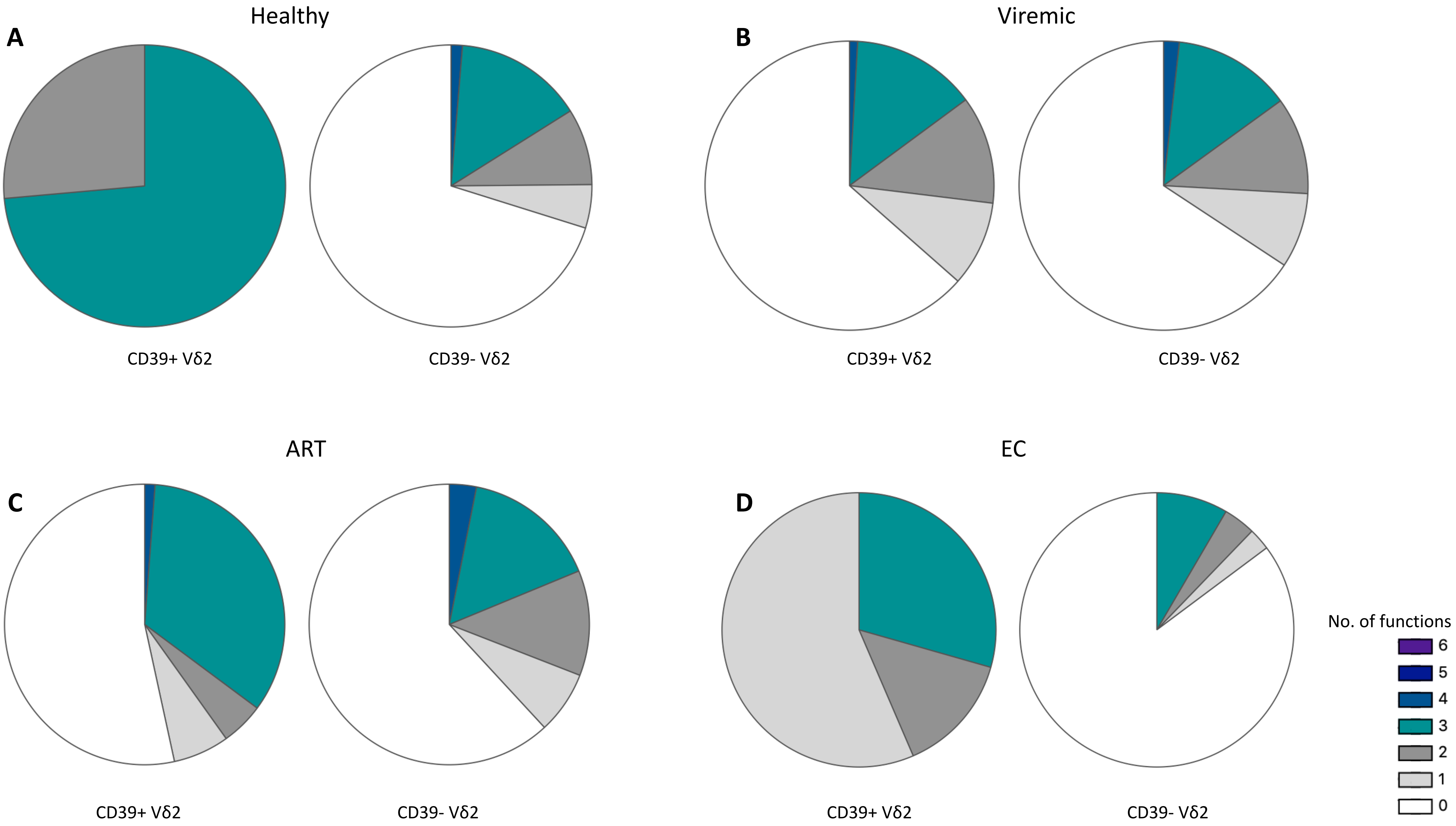
Healthy
A
CD39+ Vδ2
CD39- Vδ2
Viremic
B
CD39+ Vδ2
CD39- Vδ2
ART
C
CD39+ Vδ2
CD39- Vδ2
EC
D
CD39+ Vδ2
CD39- Vδ2
No. of functions
6
5
4
3
2
1
0

Suppl. Fig. 11
