# Supplementary material for: Inversed Ratio of CD39/CD73 Expression on γδ T Cells in HIV Versus Healthy Controls Correlates With Immune Activation and Disease Progression
Source: Front Immunol. 2022 Apr 22;13:867167. doi: 10.3389/fimmu.2022.867167 (PMC9074873; doi:10.3389/fimmu.2022.867167)
Supplement: Supplementary Table 1 — Basic demographic and virologic data of the HBV- and HCV-infected patients (average, min - max). [file Table_1.pdf]

Supplemental Table 1. Basic demographic and virologic data of the HBV- and HCV-infected patients (average, min - max).

	HBV acute	***HBV chronic***	***HCV chronic***
n	**3**	**3**	**5**
Age (years)	**30**<br>21 – 49	**36**<br>25 - 50	**51**<br>40 - 63
Viral load (IU/mL)	**235441333**<br>24000 - 700000000	**304**<br>22 - 510	**520009**<br>15 - 1980000
