# Supplementary material for: Inversed Ratio of CD39/CD73 Expression on γδ T Cells in HIV Versus Healthy Controls Correlates With Immune Activation and Disease Progression
Source: Front Immunol. 2022 Apr 22;13:867167. doi: 10.3389/fimmu.2022.867167 (PMC9074873; doi:10.3389/fimmu.2022.867167)
Supplement: Supplementary Table 2 — Overview of fluorochrome-conjugated antibodies used for phenotypic characterization via flow cytometry (surface staining). [file Table_2.pdf]

Supplemental Table 2. Overview of fluorochrome-conjugated antibodies used for phenotypic characterization via flow cytometry (surface staining).

Fluorochrome	*Antigen*	*Clone*	*Supplier*
BUV737	CD45RA	HI100	BD
BUV395	CD4	RPA-T4	BD
BV785	HLA-DR	L243	BioLegend
BV711	CD27	M-T271	BioLegend
BV650	CD279 (PD-1)	EH12.2H7	BioLegend
BV605	TIGIT	A15153G	BioLegend
BV510	CD8	SK1	BioLegend
BV421	CD28	CD28.2	BioLegend
FITC	Vδ2	IMMU389	Beckman Coulter
PE-Cy7	CD39	A1	BioLegend
PE-Texas-Red	CD38	HIT2	BioLegend
PE	Pan γδ	11F2	BD
APC-Cy7	CD19	HIB19	BioLegend
APC-Cy7	CD14	63D3	BioLegend
APC	CD73	AD2	BioLegend
Alexa Fluor 700	CD3	SK7	BioLegend
