# Supplementary material for: Inversed Ratio of CD39/CD73 Expression on γδ T Cells in HIV Versus Healthy Controls Correlates With Immune Activation and Disease Progression
Source: Front Immunol. 2022 Apr 22;13:867167. doi: 10.3389/fimmu.2022.867167 (PMC9074873; doi:10.3389/fimmu.2022.867167)
Supplement: Supplementary Table 3 — Overview of fluorochrome-conjugated antibodies used for functional characterization via flow cytometry (intracellular cytokine staining). [file Table_3.pdf]

Supplemental Table 3. Overview of fluorochrome-conjugated antibodies used for functional characterization via flow cytometry (intracellular cytokine staining).

Fluorochrome	***Antigen***	***Clone***	***Supplier***
BUV737	IL-2	MQ1-17H12	BD
BUV395	CD4	RPA-T4	BD
BV785	IFN-γ	4S.B3	BioLegend
BV711	TNF-α	MAb11	BioLegend
BV650	IL-10	JES3-9D7	BD
BV605	TIGIT	A15153G	BioLegend
BV510	CD8	SK1	BioLegend
BV421	CD28	CD28.2	BioLegend
PerCP-Cy5-5	TGF-beta	TW4-2F8	BioLegend
FITC	Vδ2	IMMU389	Beckman Coulter
PE-Cy7	CD39	A1	BioLegend
PE-Texas-Red	Granzyme B	QA16A02	BioLegend
PE	Pan γδ	11F2	BD
APC-Cy7	CD19	HIB19	BioLegend
APC-Cy7	CD14	63D3	BioLegend
APC	CD73	AD2	BioLegend
Alexa Fluor 700	CD3	SK7	BioLegend
